# ICG fluorescence imaging-guided bile leak detection to reduce clinically relevant bile leakage after hepatectomy: A protocol for a systematic review and meta-analysis

**DOI:** 10.1371/journal.pone.0346089

**Published:** 2026-07-02

**Authors:** Takehiko Hanaki, Yuji Shibata, Hiroshi Sunada, Hisashi Noma, Masaru Ueki, Yoshiyuki Fujiwara

**Affiliations:** 1 Division of Gastrointestinal and Pediatric Surgery, Tottori University Faculty of Medicine, Yonago, Tottori, Japan; 2 Division of Medical Education, Tottori University Faculty of Medicine, Yonago, Tottori, Japan; 3 Department of Thoracic/Head and Neck Medical Oncology, University of Texas, M.D. Anderson Cancer Center, Houston, Texas, United States of America; 4 Department of Advanced Medicine, Innovation, and Clinical Research Centre, Tottori University Hospital, Yonago, Tottori, Japan; 5 Department of Interdisciplinary Statistical Mathematics, The Institute of Statistical Mathematics, Tachikawa, Tokyo, Japan; Athens Medical Group, Psychiko Clinic, GREECE

## Abstract

**Background:**

Bile leakage remains a clinically relevant complication after hepatectomy and contributes to morbidity, prolonged drainage, extended hospital stay, and the need for reintervention. Intraoperative indocyanine green (ICG) fluorescence imaging can be used to visualize bile leaks from the transection plane or biliary stump, enabling targeted repair. However, evidence for the effect of this technique on clinically relevant bile leakage is heterogeneous and has not been systematically synthesized.

**Methods and analysis:**

This protocol describes a systematic review and meta-analysis of randomised controlled trials and comparative nonrandomised studies evaluating indocyanine green fluorescence imaging-guided intraoperative bile leak detection during hepatectomy. MEDLINE, Embase, Cochrane CENTRAL, and the Web of Science Core Collection will be searched from inception, along with trial registries and citation tracking. The primary outcome is clinically relevant postoperative bile leakage, defined as International Study Group of Liver Surgery (ISGLS) grade B or C. Secondary outcomes include any bile leakage, bile leak-related interventions, major postoperative complications, length of postoperative hospital stay, and mortality. Randomised and nonrandomised studies will be synthesized separately. A meta-analysis will be performed when the studies are sufficiently comparable; otherwise, the findings will be summarized narratively. Planned analyses include random-effects models, subgroup analyses stratified according to the route of indocyanine green administration, sensitivity analyses, and an assessment of the certainty of evidence using the Grading of Recommendations Assessment, Development and Evaluation approach. This protocol is registered in PROSPERO (CRD420261291065). This manuscript describes the planned methods only; study selection, data extraction, and evidence synthesis results will be reported in the completed systematic review.

**Ethics and dissemination:**

This study will use data from published studies and does not require ethics approval. The findings will be disseminated through peer-reviewed publications and conference presentations.

## Introduction

Posthepatectomy bile leakage remains a clinically consequential complication, with reported incidences varying widely according to the type and extent of liver resection. Recent studies have reported bile leakage rates of approximately 10–14% after hepatectomy in general cohorts and 13.5% after laparoscopic major hepatectomy, whereas rates may exceed 30% in patients undergoing hepatectomy with hepaticojejunostomy [1–4]. In contemporary cohorts, bile leakage has been associated with substantially increased morbidity, including higher rates of infectious complications, increased need for endoscopic or percutaneous drainage, and prolonged hospitalization. For example, one recent cohort reported infectious complications in 46% of patients with bile leakage versus 8% without bile leakage, with a median hospital stay of 26 versus 7 days [1]. In laparoscopic major hepatectomy, bile leakage was associated with increased postoperative mortality (10.0% vs. 1.6%) and prolonged hospital stay (18 vs. 8 days) [3]. In patients undergoing hepatectomy with hepaticojejunostomy, bile leakage occurred in 33.4% of patients; of these, 42.5% required interventional radiology-guided drainage and 9.6% required re-exploration [2]. Severe bile leakage, particularly ISGLS grade C, has also been associated with increased risk of surgical site infection, 90-day mortality, and prolonged length of stay [4]. These clinical consequences emphasize the importance of prevention and early intraoperative detection. A conventional intraoperative leak assessment might miss subtle or multifocal leaks from the transection plane, where leakage points can be small, intermittent, and difficult to identify under white light and where test performance may vary depending on the method and conditions used [5–8].

Several intraoperative bile leak tests have been evaluated after liver resection, including saline or air injection, nonfluorescent dye injection, and the white test using fat emulsion. A previous systematic review and meta-analysis by Vaska et al. assessed intraoperative bile leak testing after liver resection in general and suggested that bile leak testing may reduce postoperative bile leakage and related postoperative outcomes [9]. However, that review was not specifically focused on indocyanine green (ICG) fluorescence imaging, did not use clinically relevant International Study Group of Liver Surgery (ISGLS) grade B/C bile leakage [10] as the primary outcome, and did not evaluate heterogeneity according to the route of ICG administration.

ICG fluorescence imaging allows the visualization of fluorescent bile under near-infrared light and can assist in lesion-directed intraoperative repair by highlighting leakage sites that might otherwise be missed [11–13]. The use of ICG fluorescence imaging for bile leak testing after hepatectomy was reported in early clinical studies, including intrabiliary administration approaches [14], and subsequent studies have evaluated ICG fluorescence-guided bile leak detection using intrabiliary or systemic administration [13,15–17]. However, clinical effectiveness is likely to vary across ICG administration routes (intravascular vs. intrabiliary), dosing and timing, imaging systems, and surgical factors, such as the extent of hepatectomy and surgical approach, as well as the study design [15,16]. Although previous reviews have addressed broader applications of ICG fluorescence imaging in liver resection, especially for tumour identification, margin assessment, and segmental demarcation [18,19], no dedicated systematic review has specifically focused on ICG-guided intraoperative bile leak detection and repair during hepatectomy. Therefore, this review will specifically evaluate the comparative effectiveness of ICG-guided intraoperative bile leak detection and repair for reducing clinically relevant postoperative bile leakage (ISGLS grade B/C) after hepatectomy. The review will also explore heterogeneity according to ICG administration route and relevant surgical factors. This manuscript reports the protocol for the planned systematic review and meta-analysis and does not include study selection results, extracted data, or synthesized findings.

## Materials and methods

### Objectives

This systematic review and meta-analysis aims to determine whether ICG fluorescence imaging-guided intraoperative bile leak detection decreases clinically relevant postoperative bile leakage after hepatectomy, defined as ISGLS grade ≥B, compared with a conventional assessment without ICG [10]. The secondary aims are to evaluate the effects of ICG imaging on any bile leakage, bile leak-related interventions, major postoperative complications, length of postoperative hospital stay, and mortality and to explore heterogeneity across ICG administration routes.

### Protocol and reporting standards

This protocol has been prepared in accordance with the PRISMA-P guidelines [20] (see S1 Checklist). The final systematic review will be reported according to PRISMA 2020 [21]. The protocol has been registered in PROSPERO (CRD420261291065). Any amendments will be documented with rationale and date-stamped updates in the PROSPERO record and described in the final manuscript.

### Eligibility criteria

#### Population.

The study population will comprise patients undergoing hepatectomy by any approach, including open, laparoscopic, or robotic surgery. For this review, hepatectomy will be operationally defined as surgical resection of liver parenchyma involving a transection plane, including wedge resection, partial hepatectomy, segmentectomy, sectionectomy, hemihepatectomy, and extended hepatectomy. Procedures without liver parenchymal resection, such as ablation alone or nonresectional biliary procedures, will not be considered hepatectomy.

For this review, hepatectomy-specific data will be defined as outcome data reported separately for patients undergoing liver resection in whom postoperative bile leakage is attributable to the hepatic transection plane and/or biliary stump, rather than to biliary-enteric anastomosis, pancreatic surgery, or other non-hepatectomy procedures.

Studies enrolling mixed hepatobiliary or mixed surgical populations will be eligible for primary quantitative synthesis only when outcome data for eligible hepatectomy patients without biliary reconstruction are separately reported or can be derived from the publication. If hepatectomy-specific outcome data cannot be separated, the study will not be included in the primary quantitative synthesis. We will not apply a proportion-based threshold for nonseparable mixed cohorts; instead, separability of hepatectomy-specific outcomes will be required for inclusion in quantitative synthesis. Such studies may be summarized narratively only when they provide relevant contextual information on ICG fluorescence imaging-guided bile leak detection.

### Exclusion criteria

Liver transplantation cohorts; paediatric-only cohorts; studies in which hepatectomy-specific outcomes cannot be extracted; and studies in which postoperative bile leakage mainly results from biliary reconstruction or biliary–enteric anastomosis (such as hepaticojejunostomy/choledochojejunostomy or extrahepatic bile duct resection) will be excluded from the primary quantitative synthesis. Studies focused on pancreatic surgery or hepaticojejunostomy-related leakage will be used only as background literature or narrative context, where relevant, and will not be eligible for quantitative synthesis of bile leakage from the hepatic transection plane or biliary stump after hepatectomy.

### Intervention

Intraoperative ICG fluorescence imaging is specifically used to detect bile leaks and guide immediate repair during hepatectomy, focusing on the hepatectomy transection plane and/or biliary stump, regardless of the ICG administration route (intravascular and/or intrabiliary injection) and the fluorescence imaging system (i.e., any near-infrared fluorescence platform).

**Operational definition**: The report explicitly describes (1) preoperative and/or intraoperative ICG administration via an intravascular route (venous or portal venous) and/or via an intrabiliary route, with near-infrared fluorescence visualization of ICG-containing bile and (2) the use of fluorescence findings to identify suspected bile leakage sites and guide intraoperative management, including targeted suturing, clipping, sealing, or other forms of repair/reinforcement.

**Exclusion by intent**: Studies will be excluded when ICG fluorescence imaging is used only for non-bile leak purposes—including tumour localization/navigation, segmental delineation, perfusion assessment, or vascular imaging—without an explicit intent to detect bile leakage or guide repair. Studies will not be excluded when bile leak detection is an explicit component of the fluorescence-guided intraoperative strategy.

Primary intent of ICG use: For included studies, the primary intent of ICG administration will be classified as follows: (1) primary bile leak detection, in which ICG is administered specifically to detect bile leakage and guide intraoperative repair; (2) multipurpose or other primary fluorescence-guided intent, in which ICG is administered primarily for another purpose, such as tumour localization, segmental demarcation, anatomical resection, perfusion assessment, or vascular imaging, but bile leak detection is explicitly described as a component of the intraoperative strategy; and (3) unclear intent, when the primary intent cannot be determined from the report. Studies without any explicit intent to detect bile leakage or guide repair will be excluded.

### Definitions of ICG administration routes (operational)

We will categorize ICG administration for bile leak detection based on the administration route and specific technique used.

A. Intravascular (systemic venous and/or portal venous) administration (IV)
  - ICG is delivered via systemic venous access and/or through the portal venous system, aiming for it to be excreted into bile and visualized intraoperatively using near-infrared fluorescence imaging.
  - Representative techniques include intravenous bolus injection, intravenous infusion, intraportal (portal venous) injection, and single or repeated dosing.
  - The timing and dose of ICG administration will be recorded, including preoperative dosing (i.e., hours to days before surgery) or intraoperative dosing (i.e., at skin incision, at the start of parenchymal transection, during transection, or after transection).
B. Intrabiliary administration (IB)
  - ICG is administered directly into the biliary tree to locally label bile, which is then visualized through fluorescence.
  - We subclassify the technique as follows:
    - IB-1 (via the cystic duct/gallbladder/biliary stump cannulation): ICG is injected through a cannulated cystic duct, gallbladder, or biliary stump during surgery.
    - IB-2 (transpapillary endoscopic route): ICG is injected via a transpapillary biliary catheter placed pre- or intraoperatively.
    - IB-3 (preoperatively placed percutaneous transhepatic route): ICG is injected via a percutaneous transhepatic biliary drainage catheter or via a catheter placed during percutaneous transhepatic cholangiography.
    - IB-4 (direct intraoperative puncture/needle injection): ICG is directly injected into the bile duct under direct vision (when clearly described), including intraoperative transhepatic puncture with intrabiliary injection when a percutaneous transhepatic access route is created de novo during surgery.
C. Mixed/combined administration (MIXED)
  - Studies involving both intravascular and intrabiliary ICG administration within the same intervention protocol (either sequentially or concurrently) will be classified as MIXED.
D. Unclear/insufficiently described administration route (UNCLEAR)
  - If the administration route cannot be identified from the report (i.e., it is insufficiently described), we will classify it as UNCLEAR and attempt to contact the corresponding author. If the issue remains unresolved, the study will be retained in the main synthesis but excluded from administration route-based subgroup analyses.

### Comparators

A conventional intraoperative bile leak assessment performed without ICG fluorescence imaging, namely, the surgeon’s judgement under white light, with or without additional leak tests (such as saline or air injection, nonfluorescent dye injection, or the “white test” using fat emulsion), or standard care as defined by each study, will be used as the comparator.

### Study design

We will include randomised controlled trials and comparative nonrandomised studies (prospective or retrospective) that evaluate ICG fluorescence imaging-guided intraoperative bile leak detection and compare the results with a control group without ICG fluorescence imaging. The included studies must report outcomes for both groups to enable a comparative estimation of the effects. Single-arm case series and other noncomparative designs will be excluded from the quantitative synthesis; when relevant, they will be summarized narratively to describe the evidence base.

### Context

Any hospital setting and country.

### Information sources and search strategy

Electronic searches will be conducted in MEDLINE (via PubMed), Embase, Cochrane CENTRAL, and Web of Science Core Collection from inception to the date of the final search. We will also search ClinicalTrials.gov and the WHO International Clinical Trials Registry Platform (ICTRP), screen the reference lists of the included studies and relevant reviews, and conduct forward and backward citation tracking. No language or date restrictions will be applied during the search process, and no study design filter will be used. Articles published in a language other than English will be translated as needed (e.g., by native speakers or using professional services when available), and key data items will be verified by a second reviewer. Full search strategies for all databases and registries are provided in S2 Appendix.

### Study records

#### Data management.

Records retrieved from each source will be exported and imported into R (R Foundation for Statistical Computing, Vienna, Austria) for data cleaning, harmonization, and deduplication. Deduplication will be performed using procedures based on unique identifiers and bibliographic fields, such as DOI and PMID matching, supplemented by title-based algorithms and manual verification of uncertain pairs. The deduplicated records will then be imported into a systematic review management platform to facilitate screening, maintain audit trails (including reasons for exclusion), and support data extraction.

#### Selection process and data extraction.

Two reviewers (TH and YS) will independently screen titles, abstracts and full texts in duplicate using the eligibility criteria and will extract data in duplicate using a piloted, standardized extraction form. Reasons for exclusion at the full-text stage will be documented in the review management platform, that is, recorded as prespecified exclusion categories. Disagreements will be resolved through discussion and consensus; if unresolved, a third reviewer (YF or MU) will adjudicate. Extracted items will include: study characteristics (design, setting, and enrolment period); participant characteristics; surgical factors (extent of hepatectomy, anatomical versus non-anatomical resection when reported, and operative approach); intervention characteristics (ICG administration route, dose, concentration, timing, fluorescence imaging platform, and intraoperative decision rules); comparator characteristics (intraoperative leak assessment method); whether therapeutic intervention or treatment escalation was explicitly attributed to bile leakage; whether the intervention went beyond routine postoperative care or prophylactic drainage; follow-up windows; and outcome data (event counts and denominators). For comparative nonrandomised studies, adjusted effect estimates, the effect measure used, and the covariates included in the adjustment model will be extracted when available. We will also extract information on prespecified confounding domains, including the surgical period, surgeon or institutional experience, institutional perioperative practice including enhanced recovery pathways and drain management policy, extent of hepatectomy, surgical approach, background liver disease or cirrhosis, cholestasis or biliary obstruction, biliary reconstruction or extrahepatic bile duct resection, surgical indication or tumour type, and history of preoperative chemotherapy. Co-interventions used for the intraoperative assessment of bile leaks (i.e., white test, saline/air injection, and nonfluorescent dye tests) will be extracted and documented. Studies that use ICG fluorescence imaging alongside conventional leak tests will be categorized into the ICG group, as the main comparison is ICG use versus no ICG use. HS and HN will provide statistical input for prespecified synthesis methods, subgroup and sensitivity analyses, and the interpretation of quantitative results. When the necessary data are missing or not fully reported, we will contact the authors of the study, if possible, to request additional information. We will prioritize the extraction of intention-to-treat estimates when available and address or resolve unit-of-analysis issues (patients vs. procedures) to prevent double-counting in the meta-analysis.

Potential patient or institutional cohort overlap among multiple reports from the same or potentially overlapping cohort will be assessed during study selection and data extraction. We will compare the institution, author group, enrolment period, study design, intervention protocol, sample size, ethics approval or trial registration information, and patient characteristics. The possibility of cohort overlap and the rationale for selecting one report over another will be recorded in the data extraction form. When multiple reports are judged to include overlapping patients or institutional cohorts, the most complete comparative dataset that is most appropriate for the outcome of interest will be prioritized for quantitative synthesis. Protocols, case reports, video reports, and overlapping descriptive reports will be used only for background information or narrative description, where relevant, and will not be double-counted in any meta-analysis.

### Outcomes

#### Primary outcome.

The primary outcome is clinically relevant postoperative bile leakage, defined as ISGLS grade B or C (grade ≥B) [10].

Mapping rule (when ISGLS grading is not reported): Non-ISGLS outcome definitions will be mapped to Grade ≥B-equivalent only when the report explicitly states that therapeutic intervention or treatment escalation was required or performed because of postoperative bile leakage and that the intervention went beyond routine postoperative care or prophylactic drainage. Eligible indicators include therapeutic ERCP, percutaneous drainage added, exchanged, or reinserted for the treatment of bile leakage, reoperation, antibiotic initiation or modification for bile leakage, fasting or enteral/parenteral nutritional support due to bile leakage, clearly prolonged drainage due to bile leakage, prolonged hospitalization due to bile leakage, or other active treatment specifically attributed to bile leakage. Routine drain placement, prophylactic drainage, radiological detection without a change in management, or persistent drainage without explicit therapeutic intent will not be mapped as Grade ≥B-equivalent. If therapeutic intent or attribution to bile leakage cannot be determined because of insufficient reporting, the study will be excluded from the meta-analysis of the primary outcome but may still contribute to the analysis of the secondary outcomes or narrative synthesis where applicable. When multiple follow-up time points are reported for bile leakage, we will use the longest follow-up within 90 days postoperatively; if no time point falls within 90 days, we will use the time point closest to 90 days and record the follow-up window. For studies reporting shorter or variable follow-up windows, such as in-hospital, 30-day, or 90-day outcomes, the exact follow-up window will be extracted and considered when assessing clinical and methodological heterogeneity. Studies with unclear or insufficiently reported follow-up windows will be included in the primary outcome meta-analysis only when the timing of outcome ascertainment is judged to be clinically comparable to that of other included studies; otherwise, they will be summarized narratively or examined in sensitivity analyses.

#### Secondary outcomes.

- Any bile leakage (all grades/definitions as reported in each study) will be analysed as a secondary/exploratory outcome; quantitative synthesis will be performed only when outcome definitions and data structures are sufficiently comparable; otherwise, the findings will be narratively synthesized.
- Bile leak-related interventions (i.e., ERCP, percutaneous drainage, reoperation)
- Length of postoperative hospital stay
- Mortality (as reported)
- Major postoperative complications (i.e., Clavien–Dindo [22] ≥III, if reported)

### Risk of bias assessment

Randomised trials will be evaluated using RoB 2 [23]. Comparative nonrandomised studies will be evaluated using ROBINS-I V2 [40, 41], following a prespecified target trial framework. ROBINS-I V2 was selected because the review question concerns the comparative effect of an intraoperative intervention, namely ICG fluorescence imaging-guided bile leak detection, rather than the effect of an exposure. The target trial will be conceptualized as a comparison between intraoperative ICG fluorescence imaging-guided bile leak detection and conventional intraoperative assessment without ICG fluorescence imaging among patients undergoing hepatectomy. The minimum required confounding domains for nonrandomised studies will include surgical period, surgeon or institutional experience, institutional perioperative practice including enhanced recovery pathways and drain management policy, extent of hepatectomy, surgical approach, background liver disease or cirrhosis, cholestasis or biliary obstruction, biliary reconstruction or extrahepatic bile duct resection, surgical indication or tumour type, and history of preoperative chemotherapy. Co-interventions related to intraoperative bile leak assessment, including the white test, saline or air injection, and nonfluorescent dye tests, will also be considered during risk-of-bias assessment. Two reviewers will independently assess the risk of bias; disagreements will be resolved through consensus or by consulting a third reviewer (YF or MU).

### Data synthesis

We will conduct a meta-analysis when at least two studies are sufficiently comparable. Randomised controlled trials (RCTs) and comparative nonrandomised studies (NRSI; prospective or retrospective) will be synthesized in separate primary meta-analyses because of systematic differences in confounding structures and the risk of bias [24,25]. When both evidence streams are available for an outcome, we will present pooled estimates for RCTs and NRSI side by side. Any pooled analysis that combines RCTs and NRSI will be considered exploratory and reported only as a secondary analysis with cautious interpretation [24,26].

For dichotomous outcomes, the risk ratio (RR) will be used as the effect measure. A random-effects meta-analysis will be used as the primary approach. Between-study variance (τ²) will be estimated using the restricted maximum likelihood (REML) method [27], and 95% confidence intervals (CIs) for the summary risk ratios will be calculated using the Hartung–Knapp–Sidik–Jonkman method where applicable [28]. Statistical heterogeneity will be assessed using τ² and I² statistics [29,30]. When a sufficient number of studies are available, heterogeneity and effect modification will be explored through subgroup analyses, as outlined in the next section. For outcomes with limited data, we will use prespecified methods for zero-event studies. When a study reports zero events in one arm, we will apply a Haldane-type correction (default: 0.5 added to each cell) to facilitate RR estimation [31]. Studies with zero events in both arms provide no information for relative effect estimates and will be excluded from RR pooling for that outcome but will be summarized descriptively [31]. For nonrandomised studies, adjusted effect estimates will be prioritized when available and synthesized using the generic inverse variance approach. Adjusted odds ratios, risk ratios, and hazard ratios will be extracted on the logarithmic scale with their standard errors when reported or calculable. Because odds ratios, risk ratios, and hazard ratios represent different effect measures, they will not be combined in the same pooled analysis unless a clinically and statistically justified conversion is possible. If multiple adjusted models are reported, we will prioritize the model that best accounts for the prespecified confounding domains. Adjusted and unadjusted estimates will not be pooled together in the same primary synthesis. When adjusted estimates are unavailable, unadjusted event data will be analysed separately as an exploratory or sensitivity analysis, and the limitations of residual confounding will be explicitly considered [32].

For continuous outcomes, including the length of postoperative hospital stay, we will use the mean difference (MD) as the effect measure when outcomes are reported on a common scale. If outcomes are reported on different scales or are not directly comparable, quantitative synthesis will not be performed, and the results will be summarized narratively. For studies reporting medians with interquartile ranges or ranges without means and standard deviations, we will preferentially request additional data from the authors. If such data are unavailable, we will consider estimating means and standard deviations using established methods [33,34] and will assess the robustness of findings in sensitivity analyses by excluding studies that required such conversions.

Where quantitative synthesis is not appropriate because of substantial clinical or methodological heterogeneity, incompatible outcome definitions, or incomplete reporting that precludes estimation of the effect, findings will be summarized narratively and presented in structured tables [35,36]. Following SWiM guidance, studies will be grouped by outcome, study design, ICG administration route, primary intent of ICG administration, and clinically relevant surgical factors when applicable. For each outcome, we will summarize the direction and magnitude of effects, available effect estimates or event risks, confidence intervals where reported, risk of bias, and consistency across studies. For the primary outcome (clinically relevant bile leakage, ISGLS grade ≥B), quantitative pooling will be performed only when at least two studies are sufficiently comparable in design, population, and outcome definition. If pooling is not feasible, conclusions will be based on the consistency, direction, magnitude, and certainty of the available evidence rather than on statistical significance alone. Statistical analyses will be conducted using R, primarily with the metafor and meta packages [37].

### Subgroup analyses

Subgroup analyses will focus mainly on the primary outcome and key secondary outcomes for which a sufficient number of studies are available. The prespecified subgroup variables are listed below. The findings from the subgroup analyses will be interpreted cautiously owing to limited statistical power and potential residual confounding.

- **Primary intent of ICG administration**: primary bile leak detection vs. multipurpose or other primary fluorescence-guided intent with explicit bile leak assessment. Studies with unclear intent will be excluded from this subgroup analysis and summarized descriptively.
- **ICG administration route**: intravascular vs. intrabiliary injection.

Studies using both routes within the same intervention protocol will be analysed as a separate MIXED subgroup when the data allow; studies with unclear routes will be excluded from the administration route-based subgroup analyses.

- **Exploratory subgroups** (if reporting permits):
  – Surgical approach (open vs. minimally invasive). When data permit, minimally invasive surgery will be further categorized as pure laparoscopic, robotic, or robot-assisted/hybrid approaches. These categories will be examined descriptively and, if sufficient comparative data are available, in exploratory subgroup analyses.
  – Extent of hepatectomy (major vs. minor). For the purpose of subgroup analysis in this review, major hepatectomy will be defined as resection of three or more Couinaud segments, and minor hepatectomy will be defined as resection of fewer than three Couinaud segments.
  – Resection type (anatomical vs. non-anatomical/parenchymal-sparing resection, when reported). Studies in which the resection type is unclear will be summarized descriptively and excluded from this subgroup analysis.

These exploratory analyses will be interpreted carefully, recognizing the limited power and possible residual confounding [38,39].

### Sensitivity analyses

- Excluding studies at serious/critical risk of bias (ROBINS-I V2) [40,41];
- Preferentially pooling adjusted estimates for nonrandomised studies when available [32];
- Excluding studies without feasible ISGLS grade [10] ≥B mapping for the primary outcome;
- Restricting the primary analysis to cohorts in which hepatectomy without extrahepatic bile duct resection constitutes the primary surgical population, excluding studies or subgroups with extrahepatic bile duct resection when separable.

### Assessment of reporting bias

If at least 10 studies are available for an outcome, we will construct funnel plots and consider statistical tests for small-study effects while acknowledging limited power [42,43]. If fewer than 10 studies are available, funnel plots and statistical tests for small-study effects will not be performed because of insufficient power and unreliable interpretation. In such cases, reporting bias will be assessed qualitatively by considering study size, direction and magnitude of effects, trial registry records, conference abstracts, unpublished or ongoing studies, and discrepancies between protocols or registrations and published reports, where available. Funnel plots will be included as supplementary figures when applicable.

### Certainty assessment

The certainty of evidence for key outcomes will be assessed using GRADE [44].

### Guarantor

TH will act as the guarantor of the review.

### Ethics and dissemination

This study does not involve human participants and uses data from published studies; therefore, ethical approval is not necessary. The findings will be shared through peer-reviewed publications and conference presentations.

## Discussion

This protocol details a systematic review and meta-analysis to evaluate whether intraoperative ICG fluorescence imaging-guided bile leak detection reduces clinically relevant postoperative bile leakage after hepatectomy. Although ICG fluorescence imaging is being increasingly used in hepatobiliary surgery, evidence on the clinical effectiveness of bile leak detection and repair guidance is heterogeneous across administration routes, intervention techniques, and study designs.

This review specifies clinically relevant bile leakage (ISGLS grade ≥B) as the primary outcome [10]. Unlike broader reviews of intraoperative bile leak tests or ICG fluorescence imaging in liver surgery, this protocol focuses specifically on studies in which ICG fluorescence imaging is used with an explicit intent to detect bile leakage and guide intraoperative repair. This focus is clinically important because subtle or multifocal leakage from the hepatic transection plane may be missed by conventional white-light assessment, and the ability of fluorescence imaging to identify such leakage may depend on administration route, imaging system, timing, and surgical context.

We anticipate clinical and methodological heterogeneity. Sources of heterogeneity may include ICG dose and timing, intravascular versus intrabiliary administration, comparator leak tests, operative approach, extent of hepatectomy, outcome definitions, and follow-up duration. Accordingly, the review will rely on prespecified eligibility criteria, structured outcome mapping, separate synthesis of randomised and nonrandomised evidence, and cautious interpretation of subgroup findings. When quantitative pooling is not appropriate, findings will be summarized narratively using structured tables. Even if the number of eligible comparative studies is small, a prespecified systematic review remains informative because it can define the available evidence base, prevent post hoc synthesis decisions, clarify whether quantitative pooling is appropriate, and identify methodological gaps for future comparative studies or trials.

Several limitations are anticipated. The number of eligible comparative studies may be small, and outcome reporting may not consistently classify bile leakage according to ISGLS grading. In addition, evidence from nonrandomised studies may be affected by residual confounding despite prespecified risk-of-bias assessments and sensitivity analyses [26,40,41]. Therefore, the final review will interpret pooled or narrative findings in relation to risk of bias, certainty of evidence, and route-specific clinical applicability [44]. By clarifying the comparative evidence base and key sources of heterogeneity, this review may help guide future trial designs and evidence-based implementation strategies for ICG fluorescence imaging-guided intraoperative bile leak detection in hepatectomy. If the final review identifies a consistent benefit, the clinical implications will be interpreted according to the route of ICG administration, certainty of evidence, feasibility, and risk of bias, rather than as a uniform recommendation for a single administration route. If the evidence is negative, inconsistent, or of low certainty, this will support the need for adequately powered, preferably multicenter prospective studies or randomised trials. Important anticipated limitations include variation in administration routes and protocols, limited dose–response data, and the possibility that the available evidence may be dominated by single-center studies.

### Patient and public involvement

Patients and the public were not involved in the design, conduct, reporting, or dissemination plans of this research.

## Supporting information

S1 Checklist PRISMA-P checklist. (PDF)

S2 Appendix Full search strategies for all databases and registries. (PDF)
