# Supplementary material for: ICG fluorescence imaging-guided bile leak detection to reduce clinically relevant bile leakage after hepatectomy: A protocol for a systematic review and meta-analysis
Source: PLoS One. 2026 Jul 2;21(7):e0346089. doi: 10.1371/journal.pone.0346089 (PMC13327111; doi:10.1371/journal.pone.0346089)
Supplement: S2 Appendix — (PDF) [file pone.0346089.s002.pdf]

S2 Appendix

Database/Registry	Search strategy	Notes
PubMed (MEDLINE)	#1 "Hepatectomy"[MeSH] OR hepatectom*[tiab] OR "liver resection"[tiab] OR "hepatic resection"[tiab] OR (liver[tiab] AND resection*[tiab]) OR "liver surgery"[tiab] OR "liver surgeries"[tiab]	Population/procedure
Search date (YYYY-MM-DD)<br>2026-02-02	#2 "Biliary Fistula"[MeSH] OR bile leak*[tiab] OR "bile leakage"[tiab] OR biliary leak*[tiab] OR "biliary leakage"[tiab] OR "biliary fistula"[tiab]	Outcome concept
	#3 "Indocyanine Green"[MeSH] OR "indocyanine green"[tiab] OR "ICG"[tiab]	ICG concept
Time coverage (from inception to)<br>Inception to 2026-02-02	#4 fluorescen*[tiab] OR "fluorescence imaging"[tiab] OR "near-infrared"[tiab] OR NIR[tiab] OR infrared[tiab] OR "near infrared"[tiab] OR "NIR fluorescence"[tiab] OR "fluorescence-guided"[tiab]	Imaging concept
	#5 #1 AND #2 AND (#3 AND #4)	Core query
	#6 Limits: none (no language/date restrictions; no study design filter)	
Embase (Elsevier Embase)	#1 'hepatectomy'/exp OR hepatectom*:ti,ab OR 'liver resection':ti,ab OR 'hepatic resection':ti,ab OR (liver:ti,ab AND resection*:ti,ab) OR 'liver surgery':ti,ab OR 'liver surgeries':ti,ab	Population/procedure (Emtree + keywords
Search date (YYYY-MM-DD)	#2 'bile leak'/exp OR 'biliary fistula'/exp OR (bile:ti,ab AND leak*:ti,ab) OR (biliary:ti,ab AND leak*:ti,ab) OR 'biliary fistula':ti,ab	Outcome (Emtree + keywords)
2026-02-02	#3 'indocyanine green'/exp OR 'indocyanine green':ti,ab OR icg:ti,ab	ICG concept
Time coverage (from inception to)<br>Inception to 2026-02-02	#4 fluorescen*:ti,ab OR 'fluorescence imaging':ti,ab OR 'near infrared':ti,ab OR 'near-infrared':ti,ab OR nir:ti,ab OR 'infrared':ti,ab OR 'NIR fluorescence':ti,ab OR 'fluorescence-guided':ti,ab	Imaging concept
	#5 #1 AND #2 AND (#3 AND #4)	Core query
	#6 Limits: none (no language/date restrictions; no study design filter)	
Cochrane CENTRAL (Cochrane Library)	#1 hepatectom* OR (liver AND resection*) OR "liver resection" OR "hepatic resection" OR "liver surgery" OR "liver surgeries"	Population/procedure
Search date (YYYY-MM-DD)	#2 bile leak* OR "bile leakage" OR biliary leak* OR "biliary leakage" OR "biliary fistula"	Outcome
2026-02-02	#3 "indocyanine green" OR ICG	ICG
Time coverage (from inception to)	#4 fluorescen* OR "fluorescence imaging" OR "near infrared" OR "near-infrared" OR NIR OR "NIR fluorescence" OR infrared	Imaging
Inception to 2026-02-02	#5 (#1 AND #2 AND #3 AND #4)	
	#6 Limits: none (no language/date restrictions; no study design filter)	Core query
Web of Science Core Collection	#1 TS=(hepatectom* OR "liver resection" OR "hepatic resection" OR (liver NEAR/2 resection*) OR "liver surgery" OR "liver surgeries")	Population/procedure
Search date (YYYY-MM-DD)	#2 TS=(bile leak* OR "bile leakage" OR biliary leak* OR "biliary leakage" OR "biliary fistula")	Outcome
2026-02-02	#3 TS=("indocyanine green" OR ICG)	ICG
Time coverage (from inception to)<br>Inception to 2026-02-02	#4 TS=(fluorescen* OR "fluorescence imaging" OR "near-infrared" OR "near infrared" OR NIR OR "NIR fluorescence" OR infrared OR "fluorescence-guided")	Imaging
	#5 #1 AND #2 AND #3 AND #4	Core query
ClinicalTrials.gov<br>Search date (YYYY-MM-DD)<br>2026-02-02<br>Time coverage (from inception to)<br>Inception to 2026-02-02	Condition or disease: (hepatectomy OR "liver resection" OR "hepatic resection" OR "liver surgery"); Other terms: ("indocyanine green" OR ICG) AND ("bile leak" OR "bile leakage" OR "biliary leak" OR "biliary leakage" OR "biliary fistula")	Keep broad; no restrictions unless neede[illegible]
WHO ICTRP<br>Search date (YYYY-MM-DD)<br>2026-02-02<br>Time coverage (from inception to)<br>Inception to 2026-02-02	Search terms: (hepatectomy OR "liver resection" OR "hepatic resection" OR "liver surgery") AND ("indocyanine green" OR ICG) AND ("bile leak" OR "bile leakage" OR "biliary leak" OR "biliary leakage" OR "biliary fistula")	ICTRP uses simple keyword fields; keep [illegible]
Other sources (if applicable)<br>Search date (YYYY-MM-DD)<br>2026-02-02		Backward and forward citation tracking of included studies and relevant reviews
