# Supplementary material for: ICG fluorescence imaging-guided bile leak detection to reduce clinically relevant bile leakage after hepatectomy: A protocol for a systematic review and meta-analysis
Source: PLoS One. 2026 Jul 2;21(7):e0346089. doi: 10.1371/journal.pone.0346089 (PMC13327111; doi:10.1371/journal.pone.0346089)
Supplement: Attachment — Submitted filename: 260612 Response to reviwers and editors.docx [file pone.0346089.s004.docx]

Thank you for considering our manuscript for publication in PLOS ONE. We are grateful for the time and effort the editors and reviewers have devoted to evaluating our work. In response to their valuable feedback, we have revised the manuscript and believe it has been substantially improved. Our point-by-point responses to all comments are provided below. (All responses in this comments sheet are shown in GREEN text.)

-----------

This decision reflects the overall merit of the work. Several reviewers accepted the manuscript without reservation, commending its PRISMA-P compliance, pre-registration, and clinical relevance. But a number of substantive methodological concerns must be addressed before the manuscript can be accepted.

The required revisions are as follows:

1. ROBINS-I should be replaced with ROBINS-E, which is the current methodological standard for this type of bias assessment.

We thank the Academic Editor for this important methodological comment. We agree that the risk-of-bias tool should be current and appropriate for the type of evidence being assessed.

Before finalizing the revision, we sought clarification from the PLOS ONE Editorial Office regarding whether ROBINS-E or an intervention-focused tool would be more appropriate for this review. We explained that the review question concerns the comparative effect of an intraoperative intervention, namely ICG fluorescence imaging-guided bile leak detection, rather than the effect of an exposure. The Associate Editor agreed that a tool designed to assess interventional studies is most appropriate and recommended specifying ROBINS-I V2 in the assessment plan.

Accordingly, we have revised the “Risk of bias assessment” section to specify that comparative nonrandomized studies will be assessed using ROBINS-I V2. We also added a brief justification in the protocol, stating that ROBINS-I V2 was selected because this review evaluates the comparative effect of an intraoperative intervention rather than an exposure. In addition, we strengthened the prespecified target trial framework, minimally required confounding domains, relevant co-interventions, and the handling of adjusted and unadjusted estimates.

2. The ISGLS mapping rule for studies not reporting standardized grading requires tightening. The current rule risks overestimating Grade B leakage by equating any drainage with therapeutic intervention. Please revise to require explicit evidence of therapeutic intent beyond routine or prophylactic drainage, and add a corresponding item to the data extraction form.

We thank the Academic Editor for this important comment. We agree that the previous mapping rule could have been interpreted too broadly, particularly if drainage was considered equivalent to ISGLS grade B leakage without clear evidence of therapeutic intent.

We have revised the “Primary outcome” section to tighten the mapping rule for studies that do not report ISGLS grading. In the revised protocol, non-ISGLS outcome definitions will be mapped to Grade ≥B-equivalent only when the report explicitly states that therapeutic intervention or treatment escalation was required or performed because of postoperative bile leakage and that the intervention went beyond routine postoperative care or prophylactic drainage. We also clarified that routine drain placement, prophylactic drainage, radiological detection without a change in management, or persistent drainage without explicit therapeutic intent will not be mapped as Grade ≥B-equivalent.

In addition, we revised the “Selection process and data extraction” section to add corresponding extraction items. We will now extract whether therapeutic intervention or treatment escalation was explicitly attributed to bile leakage and whether the intervention went beyond routine postoperative care or prophylactic drainage. If therapeutic intent or attribution to bile leakage cannot be determined because of insufficient reporting, the study will be excluded from the meta-analysis of the primary outcome but may still contribute to secondary outcome analyses or narrative synthesis where applicable.

3. The background section should be substantially revised to position the present review relative to Vaska et al. (2019, HPB), which assessed intraoperative bile leak tests after liver resection in general and represents the most directly relevant predecessor. The novelty of the present review, i.e., its exclusive focus on ICG fluorescence imaging, use of ISGLS Grade B/C as the primary outcome, and stratification by administration route, should be clearly articulated.

We thank the Academic Editor for this important comment. We revised the Introduction to position the systematic review and meta-analysis by Vaska et al. as the most directly relevant predecessor on intraoperative bile leak testing after liver resection in general. We also clarified that the present review differs from previous reviews by focusing specifically on ICG fluorescence imaging-guided bile leak detection, using clinically relevant ISGLS grade B/C bile leakage as the primary outcome, and exploring heterogeneity according to the route of ICG administration. Vaska et al. has now been added as reference [9].

4. Sakaguchi et al. (2010) and Hanaki et al. (2022, Anticancer Research) should be cited and discussed. The protocol must explicitly prespecify how potential patient or institutional cohort overlap across these studies, the Hanaki 2023 protocol, and related case reports will be identified and handled.

We thank the Academic Editor for this important comment. We revised the Introduction to cite and discuss Sakaguchi et al. as an early clinical study of ICG fluorescence-based bile leak testing after hepatectomy and Hanaki et al. 2022 in Anticancer Research as a directly relevant study evaluating ICG fluorescence-guided bile leak detection. These studies are now cited in the Introduction as references [14] and [17], respectively; the relevant sentence now cites subsequent studies of ICG fluorescence-guided bile leak detection using intrabiliary or systemic administration [13, 15-17].

We agree with the Academic Editor that potential patient or institutional cohort overlap should be prespecified. We have added a new description to the “Selection process and data extraction” section. In the revised protocol, we state that possible overlap among multiple reports from the same or potentially overlapping cohort will be assessed by comparing the institution, author group, enrolment period, study design, intervention protocol, sample size, ethics approval or trial registration information, and patient characteristics. We also state that the possibility of overlap and the rationale for selecting one report over another will be recorded in the data extraction form. When overlap is suspected, the most complete comparative dataset that is most appropriate for the outcome of interest will be prioritized for quantitative synthesis, and protocols, case reports, video reports, and overlapping descriptive reports will not be double-counted in any meta-analysis.

5. Confounding variables for nonrandomized studies must be prespecified in greater detail. The authors should identify the minimally required confounders for ROBINS-E assessment and specify how adjusted and unadjusted estimates will be handled to avoid inappropriate mixing in the same synthesis.

We thank the Academic Editor for this important comment. We have revised the Methods to prespecify the handling of confounding in comparative nonrandomized studies in greater detail.

In the “Selection process and data extraction” section, we added that adjusted effect estimates, the effect measure used, and the covariates included in the adjustment model will be extracted when available. We also added that information on prespecified confounding domains will be extracted, including surgical period, surgeon or institutional experience, institutional perioperative practice including enhanced recovery pathways and drain management policy, extent of hepatectomy, surgical approach, background liver disease or cirrhosis, cholestasis or biliary obstruction, biliary reconstruction or extrahepatic bile duct resection, surgical indication or tumor type, and history of preoperative chemotherapy.

In the “Risk of bias assessment” section, we added that the assessment of comparative nonrandomized studies will follow a prespecified target trial framework. The target trial is conceptualized as a comparison between intraoperative ICG fluorescence imaging-guided bile leak detection and conventional intraoperative assessment without ICG fluorescence imaging among patients undergoing hepatectomy. The minimally required confounding domains and relevant co-interventions are now explicitly described.

In the “Data synthesis” section, we clarified that adjusted effect estimates will be prioritized when available and synthesized using the generic inverse variance approach. We also clarified that adjusted odds ratios, risk ratios, and hazard ratios will be extracted on the logarithmic scale with their standard errors when reported or calculable. Because these effect measures are not directly interchangeable, they will not be combined in the same pooled analysis unless a clinically and statistically justified conversion is possible. We further clarified that adjusted and unadjusted estimates will not be pooled together in the same primary synthesis. When adjusted estimates are unavailable, unadjusted event data will be analyzed separately as an exploratory or sensitivity analysis, and the limitations of residual confounding will be explicitly considered.

6. The inclusion of mixed hepatobiliary and biliary reconstruction procedures requires clearer boundaries. Studies involving bile leakage from biliary anastomoses or pancreatic surgery should not be pooled with hepatic transection plane leakage studies. A concrete, quantitative decision rule for inclusion of mixed-procedure cohorts should be provided.

We thank the Academic Editor for this important comment. We agree that bile leakage from biliary-enteric anastomoses or pancreatic surgery differs mechanistically from bile leakage arising from the hepatic transection plane or biliary stump and should not be pooled with the target condition of this review.

We have revised the “Population” and “Exclusion criteria” sections to clarify the boundaries for mixed hepatobiliary and mixed surgical cohorts. In the revised protocol, hepatectomy-specific data are defined as outcome data reported separately for patients undergoing liver resection in whom postoperative bile leakage is attributable to the hepatic transection plane and/or biliary stump, rather than to biliary-enteric anastomosis, pancreatic surgery, or other non-hepatectomy procedures.

We also clarified that mixed hepatobiliary or mixed surgical cohorts will be eligible for primary quantitative synthesis only when outcome data for eligible hepatectomy patients without biliary reconstruction are separately reported or can be derived from the publication. If hepatectomy-specific outcome data cannot be separated, the study will not be included in the primary quantitative synthesis. We decided not to apply a proportion-based threshold for nonseparable mixed cohorts, because doing so could still combine mechanistically different types of bile leakage. Instead, we prespecified a stricter rule requiring separability of hepatectomy-specific outcomes for inclusion in quantitative synthesis. Studies focused on pancreatic surgery or hepaticojejunostomy-related leakage will be used only as background literature or narrative context, where relevant, and will not be eligible for quantitative synthesis of bile leakage from the hepatic transection plane or biliary stump after hepatectomy.

7. The primary intent of ICG administration (bile leak detection vs. tumor localization or anatomical resection) should be addressed as a potential source of systematic heterogeneity, with clarification or stratification proposed.

We thank the Academic Editor for this important comment. We revised the “Intervention” section to clarify how the primary intent of ICG administration will be handled. In the revised protocol, included studies will be classified as primary bile leak detection, multipurpose or other primary fluorescence-guided intent with explicit bile leak assessment, or unclear intent. Studies without any explicit intent to detect bile leakage or guide repair will be excluded.

We also revised the “Subgroup analyses” section to include primary intent of ICG administration as a prespecified source of heterogeneity. When data permit, studies will be compared according to primary bile leak detection versus multipurpose or other primary fluorescence-guided intent with explicit bile leak assessment; studies with unclear intent will be excluded from this subgroup analysis and summarized descriptively.

8. An operational definition of "hepatectomy" should be provided in the eligibility criteria, and the definition of major hepatectomy used in subgroup analyses should be prespecified.

We thank the Academic Editor for this important comment. We have revised the “Population” section of the eligibility criteria to provide an operational definition of hepatectomy. In the revised protocol, hepatectomy is defined as surgical resection of liver parenchyma involving a transection plane, including wedge resection, partial hepatectomy, segmentectomy, sectionectomy, hemihepatectomy, and extended hepatectomy. We also clarified that procedures without liver parenchymal resection, such as ablation alone or nonresectional biliary procedures, will not be considered hepatectomy.

We also revised the “Subgroup analyses” section to prespecify the definition of major hepatectomy. For the purpose of subgroup analysis in this review, major hepatectomy will be defined as resection of three or more Couinaud segments, and minor hepatectomy will be defined as resection of fewer than three Couinaud segments.

9. The manuscript should be revised for conciseness, removing repetitive passages.

We thank the Academic Editor for this helpful comment. We revised the manuscript to reduce repetitive descriptions and improve readability while retaining the methodological details required for a protocol manuscript. In particular, we shortened the Discussion by removing repeated descriptions of the primary outcome, ISGLS mapping, subgroup analyses, and separate synthesis of randomized and nonrandomized studies, which are already described in detail in the Methods. The revised Discussion now focuses more concisely on the rationale, anticipated heterogeneity, limitations, and implications of the planned review.

The following points require clarification but are of lesser priority:

• Whether laparoscopic and robotic approaches will be analyzed separately within the minimally invasive surgery subgroup.

We thank the Academic Editor for this helpful clarification. We revised the “Subgroup analyses” section to specify how minimally invasive approaches will be handled. In the revised protocol, surgical approach will first be categorized as open versus minimally invasive surgery. When data permit, minimally invasive surgery will be further categorized as pure laparoscopic, robotic, or robot-assisted/hybrid approaches. These categories will be examined descriptively and, if sufficient comparative data are available, in exploratory subgroup analyses.

• How studies with shorter or variable follow-up will be handled relative to the 90-day primary outcome window.

We thank the Academic Editor for this helpful clarification. We revised the “Primary outcome” section to clarify the handling of shorter or variable follow-up windows. When multiple follow-up time points are reported, we will use the longest follow-up within 90 days postoperatively; if no time point falls within 90 days, we will use the time point closest to 90 days. For studies reporting in-hospital, 30-day, 90-day, or otherwise variable follow-up windows, the exact follow-up window will be extracted and considered when assessing heterogeneity. Studies with unclear or insufficiently reported follow-up windows will be included in the primary outcome meta-analysis only when outcome ascertainment is judged to be clinically comparable; otherwise, they will be summarized narratively or examined in sensitivity analyses.

• How publication bias will be assessed if fewer than 10 studies are available for pooling.

We thank the Academic Editor for this helpful clarification. We revised the “Assessment of reporting bias” section to clarify how reporting bias will be assessed when fewer than 10 studies are available for pooling. We now state that funnel plots and statistical tests for small-study effects will not be performed in such cases because of insufficient power and unreliable interpretation. Instead, reporting bias will be assessed qualitatively by considering study size, direction and magnitude of effects, trial registry records, conference abstracts, unpublished or ongoing studies, and discrepancies between protocols or registrations and published reports, where available.

• The narrative synthesis approach should be described in greater methodological detail, with reference to structured guidance such as SWiM.

We thank the Academic Editor for this helpful clarification. We revised the “Data synthesis” section to describe the planned narrative synthesis in greater methodological detail while keeping the protocol concise. Following SWiM guidance, studies will be grouped by outcome, study design, ICG administration route, primary intent of ICG administration, and relevant surgical factors when applicable. For each outcome, we will summarize the direction and magnitude of effects, available effect estimates or event risks, confidence intervals where reported, risk of bias, and consistency across studies. If quantitative pooling is not feasible for the primary outcome, conclusions will be based on the consistency, direction, magnitude, and certainty of the available evidence rather than on statistical significance alone.

-----------

Review Comments to the Author

Reviewer #1: This study protocol describes a systematic review and meta-analysis to evaluate the effectiveness of indocyanine green (ICG) fluorescence imaging-guided intraoperative bile leak detection in reducing clinically relevant bile leakage after hepatectomy. Post-hepatectomy bile leakage remains a significant complication associated with increased morbidity and healthcare resource utilization, making this evidence synthesis timely and clinically important. The protocol is prepared in strict accordance with the PRISMA-P guidelines, which ensures a high standard of transparency and methodological rigor.

Study Design and Eligibility: The PICO framework is explicitly defined. A notable strength is the detailed classification of ICG administration routes (intravascular, intrabiliary, and mixed), which will allow for a sophisticated assessment of clinical heterogeneity.

Outcome Definitions: The primary outcome is clearly defined as clinically relevant postoperative bile leakage (ISGLS grade B or C). The inclusion of a predefined "mapping rule" to translate non-standardized reports into ISGLS-equivalent grades is a meticulous detail that will enhance the consistency and reliability of the data synthesis.

The protocol is exceptionally well-developed, and the proposed methodology and analysis plans are comprehensive. No major flaws or significant omissions were identified. The study is well-positioned to provide high-quality evidence regarding the intraoperative use of ICG fluorescence for bile leak detection.

We sincerely thank the reviewer for the positive evaluation of our protocol and for recognizing the clinical relevance, PRISMA-P-based structure, detailed eligibility criteria, and prespecified outcome definitions. We appreciate the reviewer’s encouraging comments.

Reviewer #2: This is an excellent, well-written article with a highly appropriate methodological design. The only area for improvement concerns the risk of bias tool. Instead of ROBINS-I, which is no longer the standard practice for this type of assessment, I suggest updating the analysis to use the ROBINS-E tool. Addressing this minor point will further strengthen the paper.

We thank the reviewer for the positive assessment of our manuscript and for this helpful methodological suggestion. We agree that the risk-of-bias assessment plan should be updated and clearly justified.

Before finalizing the revision, we sought clarification from the PLOS ONE Editorial Office regarding whether ROBINS-E or an intervention-focused tool would be more appropriate for this review. We explained that the review question concerns the comparative effect of an intraoperative intervention, namely ICG fluorescence imaging-guided bile leak detection, rather than the effect of an exposure. The Associate Editor agreed that a tool designed to assess interventional studies is most appropriate and recommended specifying ROBINS-I V2 in the assessment plan.

Accordingly, we have revised the “Risk of bias assessment” section to specify that comparative nonrandomized studies will be assessed using ROBINS-I V2. We also added a brief justification in the protocol, stating that ROBINS-I V2 was selected because this review evaluates the comparative effect of an intraoperative intervention rather than an exposure.

Reviewer #3: This protocol describes a systematic review and meta-analysis evaluating whether intraoperative ICG fluorescence imaging-guided bile leak detection reduces clinically relevant postoperative bile leakage (ISGLS grade B/C) after hepatectomy. The protocol is well-structured, PRISMA-P-compliant, and pre-registered with PROSPERO. The separation of RCTs and non-randomized studies in the primary synthesis, along with pre-specified subgroup analyses stratified by ICG administration route, reflects sound methodological judgment. The protocol includes all surgical approaches and partially permits mixed hepatobiliary procedures, but three methodological concerns arise. These relate to the absence of an operational definition of "hepatectomy," the differential ICG detectability across surgical approaches, and the ambiguity of inclusion criteria for mixed procedures. My comments are as follows.

(Comments)

1 ) "Hepatectomy" lacks an operational definition. Wedge resection and extended hepatectomy differ substantially in transection surface area and bile duct caliber, yet no minimum resection unit is specified. The authors should provide an operational definition of "hepatectomy" in the Eligibility criteria.

We thank the reviewer for this helpful comment. We agree that hepatectomy should be operationally defined because the extent of liver resection may vary substantially across included studies.

We have revised the “Population” section to provide an explicit operational definition. In the revised protocol, hepatectomy is defined as surgical resection of liver parenchyma involving a transection plane, including wedge resection, partial hepatectomy, segmentectomy, sectionectomy, hemihepatectomy, and extended hepatectomy. We also clarified that procedures without liver parenchymal resection, such as ablation alone or nonresectional biliary procedures, will not be considered hepatectomy.

2) Surgical approaches differ in near-infrared camera specifications and working distance, which may systematically affect ICG detectability independent of any true clinical effect — a concern not adequately addressed by an exploratory subgroup analysis alone. The authors should explicitly justify the exploratory status of the surgical approach subgroup, or consider elevating it to a pre-specified primary subgroup.

We thank the reviewer for this important comment. We agree that surgical approach may influence ICG detectability through differences in camera systems, working distance, field of view, and imaging conditions.

We have revised the “Subgroup analyses” section to clarify how surgical approach will be handled. Surgical approach will first be categorized as open versus minimally invasive surgery. When data permit, minimally invasive surgery will be further categorized as pure laparoscopic, robotic, or robot-assisted/hybrid approaches. These categories will be examined descriptively and, if sufficient comparative data are available, in exploratory subgroup analyses.

We retained surgical approach as an exploratory subgroup rather than a primary subgroup because the number of eligible comparative studies is expected to be limited, and surgical approach is likely to be correlated with other factors, including ICG administration route, imaging platform, operative indication, extent of hepatectomy, and institutional practice. Therefore, subgroup findings by surgical approach will be interpreted cautiously as hypothesis-generating rather than confirmatory.

3) The inclusion criterion of "extractable hepatectomy-specific data" for mixed hepatobiliary procedures lacks a quantitative threshold, leaving inclusion decisions to the reviewer's discretion and weakening the protocol's pre-specified nature. The authors should specify a concrete decision rule for the inclusion of mixed hepatobiliary procedure cohorts.

We thank the reviewer for this important comment. We agree that the previous wording regarding “extractable hepatectomy-specific data” required a clearer prespecified decision rule.

We have revised the “Population” section to define hepatectomy-specific data as outcome data reported separately for patients undergoing liver resection in whom postoperative bile leakage is attributable to the hepatic transection plane and/or biliary stump, rather than to biliary-enteric anastomosis, pancreatic surgery, or other non-hepatectomy procedures.

We also clarified the decision rule for mixed hepatobiliary or mixed surgical populations. In the revised protocol, such studies will be eligible for primary quantitative synthesis only when outcome data for eligible hepatectomy patients without biliary reconstruction are separately reported or can be derived from the publication. If hepatectomy-specific outcome data cannot be separated, the study will not be included in the primary quantitative synthesis. We decided not to apply a proportion-based threshold for nonseparable mixed cohorts, because doing so could still combine mechanistically different types of bile leakage. Instead, we prespecified a stricter rule requiring separability of hepatectomy-specific outcomes for inclusion in quantitative synthesis. Nonseparable mixed cohorts may be summarized narratively only when they provide relevant contextual information on ICG fluorescence imaging-guided bile leak detection.

Reviewer #4: I would like to congratulate the authors on this exceptionally well-written, comprehensive, and methodologically robust protocol for a systematic review and meta-analysis. The topic is clinically important and timely, addressing a persistent source of morbidity after hepatectomy. I have a few questions and suggestions. Addressing these would help strengthen the manuscript’s clarity.

1. The protocol is clearly structured and thoughtfully designed. One point that would benefit from further clarification is how the authors plan to ensure consistency when translating non-ISGLS definitions of bile leak into ISGLS grade ≥B. Given the variability across studies, how will potential misclassification be minimized?

We thank the reviewer for this helpful comment. We agree that mapping non-ISGLS definitions to ISGLS grade ≥B requires a conservative and reproducible approach to minimize misclassification.

We have revised the “Primary outcome” section to clarify the mapping rule. In the revised protocol, non-ISGLS outcome definitions will be mapped to Grade ≥B-equivalent only when therapeutic intervention or treatment escalation is explicitly reported as being required or performed because of postoperative bile leakage and when the intervention clearly goes beyond routine postoperative care or prophylactic drainage. Eligible indicators include therapeutic ERCP, percutaneous drainage added, exchanged, or reinserted for the treatment of bile leakage, reoperation, antibiotic initiation or modification for bile leakage, fasting or enteral/parenteral nutritional support due to bile leakage, clearly prolonged drainage due to bile leakage, prolonged hospitalization due to bile leakage, or other active treatment specifically attributed to bile leakage.

We also clarified that routine drain placement, prophylactic drainage, radiological detection without a change in management, and persistent drainage without explicit therapeutic intent will not be mapped as Grade ≥B-equivalent. Furthermore, we revised the “Selection process and data extraction” section to extract whether the intervention or treatment escalation was explicitly attributed to bile leakage and whether it went beyond routine postoperative care or prophylactic drainage. If therapeutic intent or attribution to bile leakage is unclear, the study will not be included in the meta-analysis of the primary outcome.

2. The focus on clinically relevant bile leakage as the primary outcome is appropriate. Could the authors further justify the inclusion of “any bile leakage” as a secondary outcome, given the variability in definitions across studies?

We thank the reviewer for this helpful comment. We included “any bile leakage” as a secondary and exploratory outcome to capture the broader spectrum of postoperative bile leakage reported in the literature and to avoid discarding potentially relevant information from studies that do not report ISGLS grade B/C separately.

We agree that definitions of “any bile leakage” may vary substantially across studies. Therefore, the primary outcome remains clinically relevant bile leakage, defined as ISGLS grade B/C or Grade ≥B-equivalent using the prespecified mapping rule. In the revised protocol, we clarified that “any bile leakage” will be analyzed as a secondary/exploratory outcome and that quantitative synthesis will be performed only when outcome definitions and data structures are sufficiently comparable; otherwise, findings will be narratively synthesized.

3. The authors indicate that randomized and nonrandomized studies will be analyzed separately, which is appropriate. Could they clarify whether there are any circumstances under which these data might be combined? If so, how will they ensure that such exploratory analyses are interpreted cautiously and do not introduce bias?

We thank the reviewer for this helpful comment. We revised the “Data synthesis” section to clarify this point. Randomized controlled trials and comparative nonrandomized studies will be synthesized in separate primary meta-analyses because of systematic differences in confounding structures and risk of bias. When both evidence streams are available for an outcome, pooled estimates for randomized and nonrandomized studies will be presented side by side.

Any pooled analysis combining randomized and nonrandomized studies will be considered exploratory and reported only as a secondary analysis with cautious interpretation. The certainty of evidence will be assessed considering study design, risk of bias, and consistency of findings.

4. Considering the expected variability in ICG administration, it would be helpful if the authors could elaborate on how they plan to handle heterogeneity within subgroups, particularly when protocols are mixed or not clearly described.

We thank the reviewer for this helpful comment. We have clarified the handling of heterogeneity related to ICG administration in the revised protocol.

In the “Definitions of ICG administration routes” section, we prespecified operational categories for intravascular, intrabiliary, mixed/combined, and unclear administration routes. Studies involving both intravascular and intrabiliary ICG administration within the same intervention protocol will be classified as MIXED and analyzed as a separate subgroup when data allow. Studies with unclear administration routes will be classified as UNCLEAR; we will attempt to contact the corresponding authors, and if the issue remains unresolved, these studies will be excluded from administration route-based subgroup analyses and summarized descriptively.

In addition, we added the primary intent of ICG administration as a prespecified source of heterogeneity, distinguishing primary bile leak detection from multipurpose or other primary fluorescence-guided intent with explicit bile leak assessment.

5. Relatedly, since some studies may use ICG in combination with conventional leak tests, could this introduce performance bias? It would be helpful to clarify how such co-interventions will be accounted for in the analysis.

We thank the reviewer for this helpful comment. We agree that the combined use of ICG fluorescence imaging and conventional bile leak tests may introduce performance-related heterogeneity.

In the revised protocol, co-interventions used for intraoperative bile leak assessment, including the white test, saline or air injection, and nonfluorescent dye tests, will be extracted and documented. We also clarified in the “Risk of bias assessment” section that co-interventions related to intraoperative bile leak assessment will be considered during risk-of-bias assessment. These factors will therefore be accounted for in study characterization, risk-of-bias assessment, and interpretation of heterogeneity.

6. The primary outcome focuses on clinically relevant bile leakage within 90 days. Could the authors clarify how they will handle studies that report shorter or variable follow-up periods?

We thank the reviewer for this helpful comment. We revised the “Primary outcome” section to clarify this point. We will use the longest follow-up within 90 days when multiple time points are reported, or the time point closest to 90 days when no time point falls within 90 days. The exact follow-up window will be extracted for studies with shorter or variable follow-up. Studies with unclear or insufficiently reported follow-up windows will be included in the primary outcome meta-analysis only when outcome ascertainment is judged to be clinically comparable; otherwise, they will be summarized narratively or examined in sensitivity analyses.

7. The authors propose using funnel plots when at least 10 studies are available. Given that this may be a relatively small evidence base, how do they plan to assess publication bias if this threshold is not reached?

We thank the reviewer for this helpful comment. We revised the “Assessment of reporting bias” section to clarify the approach when fewer than 10 studies are available. In such cases, we will not perform funnel plots or statistical tests for small-study effects because their interpretation is unreliable with a small number of studies. Instead, we will assess reporting bias qualitatively by considering study size, direction and magnitude of effects, trial registry records, conference abstracts, unpublished or ongoing studies, and discrepancies between protocols or registrations and published reports, where available.

Reviewer #5: This is a well thought out and written protocol for a systematic review to synthesize available knowledge regarding the usefulness of utilizing ICG to detect bile leaks during hepatectomy.

We thank the reviewer for the positive assessment of our protocol and for recognizing the relevance and feasibility of synthesizing the available evidence on ICG fluorescence imaging-guided bile leak detection during hepatectomy. We appreciate the reviewer’s encouraging comment.

Reviewer #6: This manuscript presents a study protocol for a systematic review and meta-analysis evaluating the effectiveness of indocyanine green (ICG) fluorescence imaging in preventing postoperative bile leakage after hepatectomy.

Postoperative bile leakage is a clinically significant complication that can lead to substantial morbidity and even mortality. Therefore, this study addresses an important and clinically relevant question.

In addition, the protocol is developed in accordance with PRISMA-P guidelines, and the prospective publication of the protocol enhances transparency and methodological rigor. Overall, the manuscript is well structured and of clear clinical interest.

I have a few minor comments that may help further strengthen the clarity and robustness of the protocol:

1. Introduction (clinical impact of bile leakage)

The Introduction provides a general overview of postoperative bile leakage; however, the clinical impact could be described in greater detail.

It would strengthen the rationale if the authors could include more specific information, supported by references, regarding:

• the association between bile leakage and postoperative mortality, and

• its impact on prolonged hospitalization and healthcare burden.

Providing quantitative context would help emphasize the importance of this study.

We appreciate the reviewer’s overall positive assessment and constructive suggestions. We have revised the Introduction to provide quantitative context regarding the incidence and clinical consequences of posthepatectomy bile leakage. Specifically, we added recent data showing that bile leakage occurs in approximately 10–14% of general hepatectomy cohorts, 13.5% after laparoscopic major hepatectomy, and more than 30% in patients undergoing hepatectomy with hepaticojejunostomy. We also added data on infectious complications, need for endoscopic or percutaneous drainage, prolonged hospital stay, and mortality. These additions are intended to clarify the clinical burden of bile leakage and strengthen the rationale for evaluating intraoperative detection methods.

2. Definition of ICG administration purpose (P7, L123–)

In the section describing the definitions of ICG administration routes, it may be important to further clarify the intended purpose of ICG use.

Specifically, studies in which ICG is administered:

• primarily for bile leak detection, versus

• for other purposes (e.g., tumor localization or negative staining for anatomical resection), with bile leak detection assessed as a secondary outcome

may differ substantially in how bile leakage is visualized.

For example, in cases involving negative staining during anatomical resection, bile leakage from the transection plane may be less detectable using ICG. This difference in intended use may systematically bias the sensitivity of bile leak detection and contribute to heterogeneity in the results.

Therefore, further clarification or stratification based on the primary intent of ICG use would strengthen the methodological consistency of the review.

We thank the reviewer for this important comment. We agree that the primary intent of ICG administration may influence the detectability of bile leakage and may contribute to systematic heterogeneity.

We have revised the “Intervention” section to clarify how the primary intent of ICG use will be handled. In the revised protocol, included studies will be classified as primary bile leak detection, multipurpose or other primary fluorescence-guided intent with explicit bile leak assessment, or unclear intent. Studies without any explicit intent to detect bile leakage or guide repair will be excluded.

We also revised the “Subgroup analyses” section to include primary intent of ICG administration as a prespecified source of heterogeneity. When data permit, studies will be compared according to primary bile leak detection versus multipurpose or other primary fluorescence-guided intent with explicit bile leak assessment. Studies with unclear intent will be excluded from this subgroup analysis and summarized descriptively.

3. Clarification of “hepatectomy-specific data” (P12, L94)

The term “hepatectomy-specific data” is somewhat unclear.

It would be helpful if the authors could explicitly define what is considered “hepatectomy-specific,” particularly in studies involving mixed surgical populations.

We thank the reviewer for this helpful comment. We have revised the “Population” section to explicitly define hepatectomy-specific data. In the revised protocol, hepatectomy-specific data are defined as outcome data reported separately for patients undergoing liver resection in whom postoperative bile leakage is attributable to the hepatic transection plane and/or biliary stump, rather than to biliary-enteric anastomosis, pancreatic surgery, or other non-hepatectomy procedures.

We also clarified that studies enrolling mixed hepatobiliary or mixed surgical populations will be eligible for primary quantitative synthesis only when outcome data for eligible hepatectomy patients without biliary reconstruction are separately reported or can be derived from the publication. If hepatectomy-specific outcome data cannot be separated, the study will not be included in the primary quantitative synthesis.

4. Surgical approach (anatomical vs non-anatomical resection) (P17, L289–)

The incidence and severity of bile leakage may differ between:

• anatomical hepatectomy (e.g., lobectomy or segmentectomy), and

• non-anatomical (parenchymal-sparing) resection.

This factor may influence the outcomes and should be considered, for example, in subgroup analyses or in the interpretation of findings.

We thank the reviewer for this helpful comment. We agree that anatomical versus non-anatomical resection may influence the incidence and severity of bile leakage and may contribute to clinical heterogeneity.

We have revised the “Selection process and data extraction” section to extract resection type, namely anatomical versus non-anatomical resection, when reported. We also revised the “Subgroup analyses” section to add resection type as an exploratory subgroup variable. When data permit, studies will be examined according to anatomical versus non-anatomical/parenchymal-sparing resection. Studies in which the resection type is unclear will be summarized descriptively and excluded from this subgroup analysis. These analyses will be interpreted cautiously because of expected limited power and possible residual confounding.

5. Details of narrative synthesis

The manuscript is generally well described; however, given the anticipated heterogeneity, it is likely that some outcomes will require narrative synthesis rather than quantitative meta-analysis.

In this context, it would improve methodological transparency if the authors could provide more detailed information on:

• how studies will be grouped,

• how the direction and magnitude of effects will be summarized, and

• how conclusions (particularly for the primary outcome) will be derived in the absence of quantitative pooling.

In addition, the use of a structured approach to narrative synthesis (e.g., following SWiM guidance or using effect direction plots) may enhance reproducibility and reduce potential interpretive bias.

We thank the reviewer for this helpful comment. We revised the “Data synthesis” section to describe the planned narrative synthesis in greater methodological detail.

Following SWiM guidance, studies will be grouped by outcome, study design, ICG administration route, primary intent of ICG administration, and relevant surgical factors when applicable. For each outcome, we will summarize the direction and magnitude of effects, available effect estimates or event risks, confidence intervals where reported, risk of bias, and consistency across studies. If quantitative pooling is not feasible for the primary outcome, conclusions will be based on the consistency, direction, magnitude, and certainty of the available evidence rather than on statistical significance alone.

In summary, this is a well-designed and clinically meaningful protocol addressing an important topic. The methodology is generally sound, and the manuscript is clearly written.

Addressing the points above would further improve clarity and methodological transparency.

I therefore recommend minor revision.

We appreciate the reviewer's comment.

Reviewer #7: I read with great interest the protocol for a systematic review by Hanaki et al. The protocol is described in detail and pertains to relevant topic of ICG guided detection of bile leaks during hepatectomy. Even though the manuscript is well written, I will be more interested in the results of this systematic review and meta-analysis. I would suggest authors to make the manuscript concise as there are lot of repetitions.

We thank the reviewer for this helpful comment. We revised the Discussion to reduce repetitive descriptions and improve readability while retaining the methodological details required for a protocol manuscript. Specifically, we shortened the Discussion by removing repeated methodological details on ISGLS outcome mapping, subgroup analyses, sensitivity analyses, and separate synthesis of randomized and nonrandomized studies, which are already described in the Methods. At the same time, we retained the rationale, anticipated sources of heterogeneity, limitations, and implications of the planned review.

Reviewer #8: The overall protocol is well-designed, logically complete, and academically feasible, but there is still room for improvement. Specific comments are as follows:

1. Study Population and Exclusion Criteria

The inclusion/exclusion boundary for mixed hepatobiliary procedures and extrahepatic bile duct resection is ambiguous, which may lead to confusion in data extraction.

We thank the reviewer for this helpful comment. We agree that the previous wording could have left ambiguity regarding mixed hepatobiliary procedures and extrahepatic bile duct resection.

We have revised the “Population” and “Exclusion criteria” sections to make the inclusion and exclusion boundaries clearer. In the revised protocol, hepatectomy-specific data are defined as outcome data reported separately for patients undergoing liver resection in whom postoperative bile leakage is attributable to the hepatic transection plane and/or biliary stump, rather than to biliary-enteric anastomosis, pancreatic surgery, or other non-hepatectomy procedures. We also clarified that studies enrolling mixed hepatobiliary or mixed surgical populations will be included in quantitative synthesis only when outcome data for eligible hepatectomy patients without biliary reconstruction are separately reported or can be derived from the publication. If such data cannot be separated, the study will not be included in the primary quantitative synthesis.

2. Definition of Intervention and Comparator

Standards for ICG dosage, concentration, and timing of administration are not unified. The methods of “conventional leak testing” in the comparator group vary widely, which may introduce heterogeneity.

We thank the reviewer for this helpful comment. We agree that variability in ICG dosage, concentration, timing, administration route, and comparator leak testing may introduce clinical and methodological heterogeneity.

We have revised the “Selection process and data extraction” section to ensure that ICG administration route, dose, concentration, timing, fluorescence imaging platform, intraoperative decision rules, and comparator leak assessment methods will be extracted. We also prespecified the handling of ICG administration route, mixed or unclear administration protocols, and primary intent of ICG use in subgroup or descriptive analyses. When studies are not sufficiently comparable because of these differences, quantitative pooling will not be performed and the findings will be summarized narratively using structured tables.

Regarding the mapping rule for “clinically relevant bile leakage” (primary outcome)

The protocol stipulates that when ISGLS grading is not reported in the original studies, “requirement for clinical intervention (e.g., ERCP, drainage, reoperation)” should be used to map to Grade ≥B. This may lead to false-positive mapping. The core of ISGLS Grade B is “necessity of active therapeutic intervention”, whereas some Grade A bile leaks (only detected radiologically without changing the clinical pathway) may also receive passive prophylactic drainage. Directly equating to “drainage” may overestimate the incidence rate.

Revise the mapping rule to “explicit description of the need for/implementation of therapeutic intervention due to bile leakage (e.g., therapeutic ERCP, percutaneous drainage, reoperation) that goes beyond routine postoperative care or prophylactic drainage”. Meanwhile, add an item in the data extraction form: “whether a causal relationship between intervention and bile leakage is clearly stated”.

We thank the reviewer for this important and specific comment. We agree that directly equating drainage with ISGLS grade B-equivalent leakage could overestimate clinically relevant bile leakage, particularly when drainage is routine, prophylactic, or not clearly performed for therapeutic management of bile leakage.

We have revised the “Primary outcome” section accordingly. The revised mapping rule now requires explicit evidence that therapeutic intervention or treatment escalation was required or performed because of postoperative bile leakage and that the intervention went beyond routine postoperative care or prophylactic drainage. Percutaneous drainage will be considered Grade ≥B-equivalent only when it was added, exchanged, or reinserted for the treatment of bile leakage. In contrast, routine drain placement, prophylactic drainage, radiological detection without a change in management, or persistent drainage without explicit therapeutic intent will not be mapped as Grade ≥B-equivalent.

We also added corresponding items to the “Selection process and data extraction” section. Specifically, we will extract whether therapeutic intervention or treatment escalation was explicitly attributed to bile leakage and whether it went beyond routine postoperative care or prophylactic drainage. If the causal relationship between bile leakage and the intervention cannot be determined because of insufficient reporting, the study will be excluded from the meta-analysis of the primary outcome but may still contribute to secondary outcome analyses or narrative synthesis where applicable.

3. Outcome Measures and Conversion Rules

The equivalent conversion rule for missing ISGLS grading is excessively broad and may lead to misclassification of outcomes.

We thank the reviewer for this important comment. We agree that an overly broad conversion rule for studies not reporting ISGLS grading could lead to misclassification of clinically relevant bile leakage.

We have revised the “Primary outcome” section to make the mapping rule more conservative. In the revised protocol, non-ISGLS outcome definitions will be mapped to Grade ≥B-equivalent only when therapeutic intervention or treatment escalation is explicitly reported as being required or performed because of postoperative bile leakage and when the intervention goes beyond routine postoperative care or prophylactic drainage. Routine drain placement, prophylactic drainage, radiological detection without a change in management, or persistent drainage without explicit therapeutic intent will not be mapped as Grade ≥B-equivalent.

We also revised the “Selection process and data extraction” section to extract whether therapeutic intervention or treatment escalation was explicitly attributed to bile leakage and whether the intervention went beyond routine postoperative care or prophylactic drainage. If therapeutic intent or attribution to bile leakage cannot be determined, the study will be excluded from the meta-analysis of the primary outcome but may still contribute to secondary outcome analyses or narrative synthesis where applicable.

Regarding subgroup analysis

Using “minimally invasive surgery (MIS)” as a subgroup is appropriate. However, the value of ICG fluorescence imaging, surgical exposure, and imaging time after ICG administration may differ substantially between laparoscopic/robotic surgery and open surgery. The current protocol does not further distinguish “total robotic”, “laparoscopic”, and “robot‑assisted” procedures within the MIS group.

In subgroup analysis, if data permit, at least distinguish “pure laparoscopic/robotic” from “open” surgery. Acknowledge in the discussion that subgroup results may have interaction effects because fluorescence imaging may have higher sensitivity under MIS conditions (close lens, low-light environment).

We thank the reviewer for this helpful comment. We agree that surgical exposure, imaging conditions, and platform-specific fluorescence systems may differ between open, laparoscopic, robotic, and robot-assisted approaches.

We have revised the “Subgroup analyses” section to clarify this point. Surgical approach will first be categorized as open versus minimally invasive surgery. When data permit, minimally invasive surgery will be further categorized as pure laparoscopic, robotic, or robot-assisted/hybrid approaches. These categories will be examined descriptively and, if sufficient comparative data are available, in exploratory subgroup analyses. We also state that exploratory subgroup analyses will be interpreted carefully, recognizing limited power and possible residual confounding.

4. Discussion Section

The clinical implications of positive/negative results and directions for future research are not pre-specified.

Add the following content: If the results are positive, routine intraoperative intrabiliary ICG administration for bile leak detection is recommended; if negative, current evidence is insufficient and large‑sample RCTs are needed. Clearly state the limitations: variations in administration routes, lack of dose–response relationship, and predominance of single‑center studies.

We thank the reviewer for this helpful comment. We have revised the Discussion to clarify how the clinical implications of positive, negative, or inconclusive findings will be interpreted in the final review.

Because this is a protocol and because the review will include different ICG administration routes, we avoided prespecifying a recommendation for routine use of a single administration route before completing the evidence synthesis. Instead, we clarified that if the final review identifies a consistent benefit, the clinical implications will be interpreted according to the route of ICG administration, certainty of evidence, feasibility, and risk of bias. We also clarified that if the evidence is negative, inconsistent, or of low certainty, this will support the need for adequately powered, preferably multicenter prospective studies or randomized trials. In addition, we added anticipated limitations, including variation in administration routes and protocols, limited dose–response data, and the possibility that the available evidence may be dominated by single-center studies.

Reviewer #9: This study is a protocol for a systematic review and meta-analysis evaluating whether intraoperative indocyanine green (ICG) fluorescence imaging-guided bile leak detection, followed by immediate repair or reinforcement, reduces clinically relevant postoperative bile leakage after hepatectomy, namely ISGLS grade B/C bile leakage. The authors plan to include randomized controlled trials and comparative nonrandomized studies. The primary outcome is ISGLS grade B/C bile leakage, and secondary outcomes include any bile leakage, bile leak-related interventions, major postoperative complications, postoperative length of hospital stay, and mortality. The planned classification of ICG administration routes into intravascular, intrabiliary, and mixed administration, the separate synthesis of RCTs and NRSI, and the use of RoB 2, ROBINS-I, and GRADE are generally appropriate.

However, the current manuscript does not sufficiently explain its originality as a synthesis focused specifically on ICG and clinically relevant bile leakage. Meta-analyses of intraoperative bile leak tests in liver resection already exist. For example, the 2019 HPB review included eight studies and reported that intraoperative bile leak testing was associated with reductions in postoperative bile leakage, overall complications, reintervention, and length of hospital stay. In addition, several classical and directly relevant studies on ICG-based bile leak detection can be identified, including Sakaguchi et al. 2010, Kaibori et al. 2011, and Hanaki et al. 2022/2025. However, at least Sakaguchi 2010, Hanaki 2022 in Anticancer Research, and Vaska 2019 in HPB do not appear to be included in the manuscript’s reference list. The background section and the explanation of novelty therefore need to be reconstructed.

Major comments

1. The relationship with previous reviews of intraoperative bile leak tests in general is insufficiently explained. Although Wang 2013 is cited, the systematic review and meta-analysis by Vaska 2019 in HPB does not appear to be cited. Vaska 2019 addressed bile leak tests after liver resection in general and included ICG and the white test among its search terms. This review should be positioned as an important direct predecessor of the present manuscript. The authors should clearly differentiate the present review by stating that previous reviews assessed bile leak tests in general, whereas the present study focuses specifically on ICG fluorescence imaging, uses ISGLS grade B/C bile leakage as the primary outcome, and evaluates effects according to the route of ICG administration.

We agree with the reviewer. We revised the Introduction to explicitly describe Vaska et al. as a previous systematic review and meta-analysis evaluating intraoperative bile leak tests after liver resection in general. We then clarified the specific novelty of the present protocol: the exclusive focus on ICG fluorescence imaging-guided bile leak detection, the use of clinically relevant ISGLS grade B/C bile leakage as the primary outcome, and planned exploration according to ICG administration route. Vaska et al. has been added as reference [9].

2. Several directly relevant studies on ICG-based bile leak detection should be addressed. Sakaguchi et al. 2010, “Bile leak test by indocyanine green fluorescence images after hepatectomy,” is a foundational paper on ICG-based bile leak testing after hepatectomy and should be mentioned in the Introduction. In addition, Hanaki et al. 2022 in Anticancer Research, “Surgical Administration of Indocyanine Green in Hepatectomy for Improved Bile Leakage Detection,” is likely to include comparative data from the authors’ own group and is important both for the background of this review and for managing potential overlapping cohorts. Although Kaibori 2011 and Hanaki 2025 are cited, the protocol should explicitly state how the authors will handle possible patient or institutional overlap among these studies, Hanaki 2022, the Hanaki 2023 protocol, and related case reports.

We agree with the reviewer. We revised the Introduction to include Sakaguchi et al. 2010 as a foundational report of ICG fluorescence-based bile leak testing after hepatectomy and Hanaki et al. 2022 in Anticancer Research as a directly relevant study from our group. Sakaguchi et al. and Hanaki et al. 2022 have now been added as references [14] and [17], respectively. The revised Introduction now describes that ICG fluorescence imaging for bile leak testing was reported in early clinical studies using intrabiliary administration and that subsequent studies have evaluated ICG fluorescence-guided bile leak detection using intrabiliary or systemic administration [13, 15-17].

In addition, we have prespecified how potential overlap among related reports will be handled. We will examine overlap by comparing the institution, author group, enrolment period, study design, intervention protocol, sample size, ethics approval or trial registration information, and patient characteristics. If multiple reports are judged to include overlapping patients or institutional cohorts, the most complete comparative dataset that is most appropriate for the outcome of interest will be used for quantitative synthesis. Protocols, case reports, video reports, and overlapping descriptive reports will be retained only for background or narrative description where relevant and will not be double-counted in any meta-analysis.

3. Confounding in nonrandomized studies requires more detailed prespecification. In nonrandomized studies, especially those using historical controls, confounding is likely to be substantial. Potential confounders include surgical period, surgeon experience, institutional practice, drain placement policy, implementation of ERAS pathways, major versus minor hepatectomy, cirrhosis, cholestasis, biliary reconstruction, surgical procedure, tumor type, and history of chemotherapy. ROBINS-I is a tool for assessing bias in comparative effectiveness estimates from nonrandomized intervention studies, and its appropriate use requires prior specification of the target trial, confounding domains, co-interventions, and the approach to selection bias assessment. The authors should not merely state that adjusted estimates will be preferred. They should specify which confounders will be considered minimally necessary, how adjusted ORs, RRs, and HRs will be handled on a common effect scale, and how they will avoid mixing adjusted and unadjusted estimates in the same synthesis.

We agree with the reviewer that confounding in comparative nonrandomized studies, particularly historical-control studies, requires more detailed prespecification. We have revised the Methods accordingly.

First, in the “Selection process and data extraction” section, we now specify that adjusted effect estimates, the effect measure used, and the covariates included in each adjustment model will be extracted. We also prespecified the confounding domains that will be extracted and considered: surgical period, surgeon or institutional experience, institutional perioperative practice including enhanced recovery pathways and drain management policy, extent of hepatectomy, surgical approach, background liver disease or cirrhosis, cholestasis or biliary obstruction, biliary reconstruction or extrahepatic bile duct resection, surgical indication or tumor type, and history of preoperative chemotherapy.

Second, in the “Risk of bias assessment” section, we now describe the target trial framework for comparative nonrandomized studies as a comparison between intraoperative ICG fluorescence imaging-guided bile leak detection and conventional intraoperative assessment without ICG fluorescence imaging among patients undergoing hepatectomy. We also explicitly state the minimally required confounding domains and relevant co-interventions to be considered during risk-of-bias assessment.

Third, in the “Data synthesis” section, we clarified that adjusted estimates will be prioritized when available and synthesized using the generic inverse variance approach. Adjusted odds ratios, risk ratios, and hazard ratios will be extracted on the logarithmic scale with their standard errors when reported or calculable. Because these effect measures are not directly interchangeable, they will not be combined in the same pooled analysis unless a clinically and statistically justified conversion is possible. We also clarified that adjusted and unadjusted estimates will not be pooled together in the same primary synthesis. When adjusted estimates are unavailable, unadjusted event data will be analyzed separately as an exploratory or sensitivity analysis, and the limitations of residual confounding will be explicitly considered.

4. The eligibility criteria for biliary reconstruction and mixed hepatobiliary or pancreatic procedures should be clarified. The manuscript focuses on bile leakage from the liver transection plane or biliary stump after hepatectomy. However, although biliary reconstruction is listed as an exclusion criterion, the protocol also states that mixed cohorts may be included in sensitivity analyses or narrative synthesis when separate data are unavailable. This compromise is understandable, but bile leakage arising from biliary reconstruction or pancreatic surgery differs in mechanism, risk profile, and diagnosis from leakage from the hepatic transection plane. Such studies should not be pooled with studies of bile leakage from the liver transection surface. One of the cited references concerns ICG use for hepaticojejunostomy in robot-assisted pancreatic surgery. This may be acceptable as background literature, but the authors should clearly state that it is not direct evidence for the target condition of this review.

We agree with the reviewer. We revised the “Population” and “Exclusion criteria” sections to clarify that the target condition of this review is postoperative bile leakage attributable to the hepatic transection plane and/or biliary stump after hepatectomy. We now explicitly define hepatectomy-specific data as outcome data reported separately for patients undergoing liver resection in whom postoperative bile leakage is attributable to the hepatic transection plane and/or biliary stump, rather than to biliary-enteric anastomosis, pancreatic surgery, or other non-hepatectomy procedures.

We also removed the previous ambiguity regarding mixed cohorts with nonseparable outcomes. In the revised protocol, mixed hepatobiliary or mixed surgical cohorts will be eligible for primary quantitative synthesis only when outcome data for eligible hepatectomy patients without biliary reconstruction are separately reported or can be derived from the publication. If hepatectomy-specific outcome data cannot be separated, the study will not be included in the primary quantitative synthesis. We further clarified that studies focused on pancreatic surgery or hepaticojejunostomy-related leakage will be used only as background literature or narrative context, where relevant, and will not be eligible for quantitative synthesis of bile leakage from the hepatic transection plane or biliary stump after hepatectomy.

Minor comments

1. If major hepatectomy is used as a subgroup variable, the authors should prespecify its definition, for example, resection of three or more sections or three or more Couinaud segments.

We thank the reviewer for this helpful comment. We have revised the “Subgroup analyses” section to prespecify the definition of major hepatectomy. For the purpose of subgroup analysis in this review, major hepatectomy will be defined as resection of three or more Couinaud segments, and minor hepatectomy will be defined as resection of fewer than three Couinaud segments.

2. The authors should clarify whether laparoscopic and robotic surgery will be grouped together as minimally invasive surgery or, if feasible, analyzed separately. Camera performance, field of view, and overlay functions for ICG imaging may differ across surgical platforms.

We thank the reviewer for this helpful comment. We revised the “Subgroup analyses” section to clarify how surgical approach will be handled. Surgical approach will first be categorized as open versus minimally invasive surgery. When data permit, minimally invasive surgery will be further categorized as pure laparoscopic, robotic, or robot-assisted/hybrid approaches. These categories will be examined descriptively and, if sufficient comparative data are available, in exploratory subgroup analyses.

Reviewer #10: Bile leakage is one of the unpleasant postoperative complications that would be avoided in liver surgery. Once bile leakage occurs after liver surgery, a great deal of mental and physical stresses would be required for both the patients and surgeons. Liver surgeons have therefore made efforts to reduce the occurrence of bile leakage, it is however difficult to make zero its morbidity. At present, the intraoperative detection of bile leakage using indocyanine green (ICG) might be innovative and the authors have continued to make improvement and reports for this technique. In this study, the authors made a plan for the systematic review and meta-analysis for the detection of bile leakage using ICG. The reviewer has an interest for this report, there are however some confirmations and questions to clear up more as below:

#1: It is certainly that there were few reports of systematic review and meta-analysis for the intraoperative detection of bile leakage using ICG. On the other hand, the number of papers on this topic itself is however not particularly large. In this restrictive topic, the reviewer doubts the necessity and meaning for systematic review and meta-analysis.

We thank the reviewer for this important comment. We agree that the number of studies specifically evaluating ICG fluorescence imaging-guided intraoperative bile leak detection after hepatectomy may be limited. However, we believe that this is precisely why a prespecified systematic review protocol is useful.

The purpose of this review is not only to perform a meta-analysis when quantitative pooling is appropriate, but also to define the available evidence base, clarify differences in ICG administration routes and study designs, standardize the handling of clinically relevant bile leakage, and identify evidence gaps for future comparative studies or trials. In the revised protocol, we clarified that quantitative pooling will be performed only when studies are sufficiently comparable; otherwise, findings will be summarized using a structured narrative synthesis.

We have revised the Discussion to clarify this rationale. The revised Discussion now states that even if the number of eligible comparative studies is small, a prespecified systematic review remains informative because it can define the available evidence base, prevent post hoc synthesis decisions, clarify whether quantitative pooling is appropriate, and identify methodological gaps for future comparative studies or trials.

Reviewer #11: This study is actually a protocol to perform a study and depicts the authors' submission for publication of their desired PRISMA platform to investigate the benefits and best practices of utilizing ICG for detecting biliary leaks for hepatic resections. The concepts are all very good however it is unclear to the reader initially that there would be no reporting of outcomes or results of their analyses. I think if the authors would clarify to make that clear they are only intending to publish their methods for this analysis, it would provide more clarity to the reader

We thank the reviewer for this helpful comment and for the positive assessment of the concept of this work. We agree that it should be clear from the outset that this manuscript is a protocol and does not report the results of the systematic review.

We have revised the Abstract and Introduction to clarify this point. The revised manuscript now states that it describes the planned methods only and that study selection, data extraction, and evidence synthesis results will be reported in the completed systematic review.
