# Supplementary material for: ICG fluorescence imaging-guided bile leak detection to reduce clinically relevant bile leakage after hepatectomy: A protocol for a systematic review and meta-analysis
Source: PLoS One. 2026 Jul 2;21(7):e0346089. doi: 10.1371/journal.pone.0346089 (PMC13327111; doi:10.1371/journal.pone.0346089)
Supplement: Peer review history

# Peer review: Decision Letter 0

**DOI:** 10.1371/journal.pone.0346089.r001

**Authors:** Athanasios Pantelis

10 Jun 2026

PONE-D-26-12531

ICG fluorescence imaging-guided bile leak detection to reduce clinically relevant bile leakage after hepatectomy: a protocol for a systematic review and meta-analysis

PLOS One

Dear Dr.  Hanaki,

Thank you for submitting your manuscript to PLOS ONE. After careful consideration, we feel that it has merit but does not fully meet PLOS ONE’s publication criteria as it currently stands. Therefore, we invite you to submit a revised version of the manuscript that addresses the points raised during the review process.

==============================

**ACADEMIC EDITOR:**

Thank you for submitting your protocol manuscript to PLOS ONE. We have now received reviews from 11 expert referees. After careful consideration of all opinions, the editorial decision is:

MAJOR REVISION

This decision reflects the overall merit of the work. Several reviewers accepted the manuscript without reservation, commending its PRISMA-P compliance, pre-registration, and clinical relevance. But a number of substantive methodological concerns must be addressed before the manuscript can be accepted.

The required revisions are as follows:

1. ROBINS-I should be replaced with ROBINS-E, which is the current methodological standard for this type of bias assessment.

2. The ISGLS mapping rule for studies not reporting standardized grading requires tightening. The current rule risks overestimating Grade B leakage by equating any drainage with therapeutic intervention. Please revise to require explicit evidence of therapeutic intent beyond routine or prophylactic drainage, and add a corresponding item to the data extraction form.

3. The background section should be substantially revised to position the present review relative to Vaska et al. (2019, HPB), which assessed intraoperative bile leak tests after liver resection in general and represents the most directly relevant predecessor. The novelty of the present review, i.e., its exclusive focus on ICG fluorescence imaging, use of ISGLS Grade B/C as the primary outcome, and stratification by administration route, should be clearly articulated.

4. Sakaguchi et al. (2010) and Hanaki et al. (2022, Anticancer Research) should be cited and discussed. The protocol must explicitly prespecify how potential patient or institutional cohort overlap across these studies, the Hanaki 2023 protocol, and related case reports will be identified and handled.

5. Confounding variables for nonrandomized studies must be prespecified in greater detail. The authors should identify the minimally required confounders for ROBINS-E assessment and specify how adjusted and unadjusted estimates will be handled to avoid inappropriate mixing in the same synthesis.

6. The inclusion of mixed hepatobiliary and biliary reconstruction procedures requires clearer boundaries. Studies involving bile leakage from biliary anastomoses or pancreatic surgery should not be pooled with hepatic transection plane leakage studies. A concrete, quantitative decision rule for inclusion of mixed-procedure cohorts should be provided.

7. The primary intent of ICG administration (bile leak detection vs. tumor localization or anatomical resection) should be addressed as a potential source of systematic heterogeneity, with clarification or stratification proposed.

8. An operational definition of "hepatectomy" should be provided in the eligibility criteria, and the definition of major hepatectomy used in subgroup analyses should be prespecified.

9. The manuscript should be revised for conciseness, removing repetitive passages.

The following points require clarification but are of lesser priority:

• Whether laparoscopic and robotic approaches will be analyzed separately within the minimally invasive surgery subgroup.

• How studies with shorter or variable follow-up will be handled relative to the 90-day primary outcome window.

• How publication bias will be assessed if fewer than 10 studies are available for pooling.

• The narrative synthesis approach should be described in greater methodological detail, with reference to structured guidance such as SWiM.

Please submit your revised manuscript along with a point-by-point response to all reviewers within 22 days. The response letter should clearly indicate where each change has been made in the manuscript.

We look forward to receiving your revision.

Sincerely,

Athanasios Pantelis, MD, MSc, FMBS, FACS

Academic Editor

PLOS ONE

==============================

Please submit your revised manuscript by Jul 25 2026 11:59PM. If you will need more time than this to complete your revisions, please reply to this message or contact the journal office at plosone@plos.org. When you're ready to submit your revision, log on to https://www.editorialmanager.com/pone/ and select the 'Submissions Needing Revision' folder to locate your manuscript file.

Please include the following items when submitting your revised manuscript:

- A letter that responds to each point raised by the academic editor and reviewer(s). You should upload this letter as a separate file labeled 'Response to Reviewers'.
- A marked-up copy of your manuscript that highlights changes made to the original version. You should upload this as a separate file labeled 'Revised Manuscript with Track Changes'.
- An unmarked version of your revised paper without tracked changes. You should upload this as a separate file labeled 'Manuscript'.

If you would like to make changes to your financial disclosure, please include your updated statement in your cover letter. Guidelines for resubmitting your figure files are available below the reviewer comments at the end of this letter.

If applicable, we recommend that you deposit your laboratory protocols in protocols.io to enhance the reproducibility of your results. Protocols.io assigns your protocol its own identifier (DOI) so that it can be cited independently in the future. For instructions see: https://journals.plos.org/plosone/s/submission-guidelines#loc-laboratory-protocols. Additionally, PLOS ONE offers an option for publishing peer-reviewed Lab Protocol articles, which describe protocols hosted on protocols.io. Read more information on sharing protocols at https://plos.org/protocols?utm_medium=editorial-email&utm_source=authorletters&utm_campaign=protocols.

As the corresponding author, your ORCID iD is verified in the submission system and will appear in the published article. PLOS supports the use of ORCID, and we encourage all coauthors to register for an ORCID iD and use it as well. Please encourage your coauthors to verify their ORCID iD within the submission system before final acceptance, as unverified ORCID iDs will not appear in the published article. *Only*  the individual author can complete the verification step; PLOS staff *cannot*  verify ORCID iDs on behalf of authors.

We look forward to receiving your revised manuscript.

Kind regards,

Athanasios G. Pantelis

Academic Editor

PLOS One

Journal Requirements:

When submitting your revision, we need you to address these additional requirements.

1.Please ensure that your manuscript meets PLOS ONE's style requirements, including those for file naming. The PLOS ONE style templates can be found at

https://journals.plos.org/plosone/s/file?id=wjVg/PLOSOne_formatting_sample_main_body.pdf and

https://journals.plos.org/plosone/s/file?id=ba62/PLOSOne_formatting_sample_title_authors_affiliations.pdf

2. Please include captions for your Supporting Information files at the end of your manuscript, and update any in-text citations to match accordingly. Please see our Supporting Information guidelines for more information: http://journals.plos.org/plosone/s/supporting-information.

3.If the reviewer comments include a recommendation to cite specific previously published works, please review and evaluate these publications to determine whether they are relevant and should be cited. There is no requirement to cite these works unless the editor has indicated otherwise.

[Note: HTML markup is below. Please do not edit.]

Reviewer's Responses to Questions

**Comments to the Author**

1. Does the manuscript provide a valid rationale for the proposed study, with clearly identified and justified research questions?

The research question outlined is expected to address a valid academic problem or topic and contribute to the base of knowledge in the field.

Reviewer #1: Yes

Reviewer #2: Yes

Reviewer #3: Yes

Reviewer #4: Yes

Reviewer #5: Yes

Reviewer #6: Partly

Reviewer #7: Yes

Reviewer #8: Partly

Reviewer #9: Partly

Reviewer #10: Partly

Reviewer #11: Partly

**********

2. Is the protocol technically sound and planned in a manner that will lead to a meaningful outcome and allow testing the stated hypotheses?

The manuscript should describe the methods in sufficient detail to prevent undisclosed flexibility in the experimental procedure or analysis pipeline, including sufficient outcome-neutral conditions (e.g. necessary controls, absence of floor or ceiling effects) to test the proposed hypotheses and a statistical power analysis where applicable. As there may be aspects of the methodology and analysis which can only be refined once the work is undertaken, authors should outline potential assumptions and explicitly describe what aspects of the proposed analyses, if any, are exploratory.

Reviewer #1: Yes

Reviewer #2: Yes

Reviewer #3: Partly

Reviewer #4: Yes

Reviewer #5: Yes

Reviewer #6: Yes

Reviewer #7: Yes

Reviewer #8: Partly

Reviewer #9: Partly

Reviewer #10: Yes

Reviewer #11: Yes

**********

3. Is the methodology feasible and described in sufficient detail to allow the work to be replicable?

Descriptions of methods and materials in the protocol should be reported in sufficient detail for another researcher to reproduce all experiments and analyses. The protocol should describe the appropriate controls, sample size calculations, and replication needed to ensure that the data are robust and reproducible.

Reviewer #1: Yes

Reviewer #2: Yes

Reviewer #3: Yes

Reviewer #4: Yes

Reviewer #5: Yes

Reviewer #6: Yes

Reviewer #7: Yes

Reviewer #8: Yes

Reviewer #9: Yes

Reviewer #10: Yes

Reviewer #11: Yes

**********

4. Have the authors described where all data underlying the findings will be made available when the study is complete?

The PLOS Data policy requires authors to make all data underlying the findings described in their manuscript fully available without restriction, with rare exception, at the time of publication. The data should be provided as part of the manuscript or its supporting information, or deposited to a public repository. For example, in addition to summary statistics, the data points behind means, medians and variance measures should be available. If there are restrictions on publicly sharing data—e.g. participant privacy or use of data from a third party—those must be specified.

Reviewer #1: Yes

Reviewer #2: Yes

Reviewer #3: Yes

Reviewer #4: Yes

Reviewer #5: Yes

Reviewer #6: Yes

Reviewer #7: Yes

Reviewer #8: Yes

Reviewer #9: Yes

Reviewer #10: Yes

Reviewer #11: Yes

**********

5. Is the manuscript presented in an intelligible fashion and written in standard English?

PLOS ONE does not copyedit accepted manuscripts, so the language in submitted articles must be clear, correct, and unambiguous. Any typographical or grammatical errors should be corrected at revision, so please note any specific errors here.

Reviewer #1: Yes

Reviewer #2: Yes

Reviewer #3: Yes

Reviewer #4: Yes

Reviewer #5: Yes

Reviewer #6: Yes

Reviewer #7: Yes

Reviewer #8: Yes

Reviewer #9: Yes

Reviewer #10: Yes

Reviewer #11: Yes

**********

6. Review Comments to the Author

Please use the space provided to explain your answers to the questions above and, if applicable, provide comments about issues authors must address before this protocol can be accepted for publication. You may also include additional comments for the author, including concerns about research or publication ethics.

You may also provide optional suggestions and comments to authors that they might find helpful in planning their study.

(Please upload your review as an attachment if it exceeds 20,000 characters)

Reviewer #1: This study protocol describes a systematic review and meta-analysis to evaluate the effectiveness of indocyanine green (ICG) fluorescence imaging-guided intraoperative bile leak detection in reducing clinically relevant bile leakage after hepatectomy. Post-hepatectomy bile leakage remains a significant complication associated with increased morbidity and healthcare resource utilization, making this evidence synthesis timely and clinically important. The protocol is prepared in strict accordance with the PRISMA-P guidelines, which ensures a high standard of transparency and methodological rigor.

Study Design and Eligibility: The PICO framework is explicitly defined. A notable strength is the detailed classification of ICG administration routes (intravascular, intrabiliary, and mixed), which will allow for a sophisticated assessment of clinical heterogeneity.

Outcome Definitions: The primary outcome is clearly defined as clinically relevant postoperative bile leakage (ISGLS grade B or C). The inclusion of a predefined "mapping rule" to translate non-standardized reports into ISGLS-equivalent grades is a meticulous detail that will enhance the consistency and reliability of the data synthesis.

The protocol is exceptionally well-developed, and the proposed methodology and analysis plans are comprehensive. No major flaws or significant omissions were identified. The study is well-positioned to provide high-quality evidence regarding the intraoperative use of ICG fluorescence for bile leak detection.

Reviewer #2: This is an excellent, well-written article with a highly appropriate methodological design. The only area for improvement concerns the risk of bias tool. Instead of ROBINS-I, which is no longer the standard practice for this type of assessment, I suggest updating the analysis to use the ROBINS-E tool. Addressing this minor point will further strengthen the paper.

Reviewer #3: This protocol describes a systematic review and meta-analysis evaluating whether intraoperative ICG fluorescence imaging-guided bile leak detection reduces clinically relevant postoperative bile leakage (ISGLS grade B/C) after hepatectomy. The protocol is well-structured, PRISMA-P-compliant, and pre-registered with PROSPERO. The separation of RCTs and non-randomized studies in the primary synthesis, along with pre-specified subgroup analyses stratified by ICG administration route, reflects sound methodological judgment. The protocol includes all surgical approaches and partially permits mixed hepatobiliary procedures, but three methodological concerns arise. These relate to the absence of an operational definition of "hepatectomy," the differential ICG detectability across surgical approaches, and the ambiguity of inclusion criteria for mixed procedures. My comments are as follows.

(Comments)

1 ) "Hepatectomy" lacks an operational definition. Wedge resection and extended hepatectomy differ substantially in transection surface area and bile duct caliber, yet no minimum resection unit is specified. The authors should provide an operational definition of "hepatectomy" in the Eligibility criteria.

2) Surgical approaches differ in near-infrared camera specifications and working distance, which may systematically affect ICG detectability independent of any true clinical effect — a concern not adequately addressed by an exploratory subgroup analysis alone. The authors should explicitly justify the exploratory status of the surgical approach subgroup, or consider elevating it to a pre-specified primary subgroup.

3) The inclusion criterion of "extractable hepatectomy-specific data" for mixed hepatobiliary procedures lacks a quantitative threshold, leaving inclusion decisions to the reviewer's discretion and weakening the protocol's pre-specified nature. The authors should specify a concrete decision rule for the inclusion of mixed hepatobiliary procedure cohorts.

Reviewer #4: I would like to congratulate the authors on this exceptionally well-written, comprehensive, and methodologically robust protocol for a systematic review and meta-analysis. The topic is clinically important and timely, addressing a persistent source of morbidity after hepatectomy. I have a few questions and suggestions. Addressing these would help strengthen the manuscript’s clarity.

1. The protocol is clearly structured and thoughtfully designed. One point that would benefit from further clarification is how the authors plan to ensure consistency when translating non-ISGLS definitions of bile leak into ISGLS grade ≥B. Given the variability across studies, how will potential misclassification be minimized?

2. The focus on clinically relevant bile leakage as the primary outcome is appropriate. Could the authors further justify the inclusion of “any bile leakage” as a secondary outcome, given the variability in definitions across studies?

3. The authors indicate that randomized and nonrandomized studies will be analyzed separately, which is appropriate. Could they clarify whether there are any circumstances under which these data might be combined? If so, how will they ensure that such exploratory analyses are interpreted cautiously and do not introduce bias?

4. Considering the expected variability in ICG administration, it would be helpful if the authors could elaborate on how they plan to handle heterogeneity within subgroups, particularly when protocols are mixed or not clearly described.

5. Relatedly, since some studies may use ICG in combination with conventional leak tests, could this introduce performance bias? It would be helpful to clarify how such co-interventions will be accounted for in the analysis.

6. The primary outcome focuses on clinically relevant bile leakage within 90 days. Could the authors clarify how they will handle studies that report shorter or variable follow-up periods?

7. The authors propose using funnel plots when at least 10 studies are available. Given that this may be a relatively small evidence base, how do they plan to assess publication bias if this threshold is not reached?

Reviewer #5: This is a well thought out and written protocol for a systematic review to synthesize available knowledge regarding the usefulness of utilizing ICG to detect bile leaks during hepatectomy.

Reviewer #6: This manuscript presents a study protocol for a systematic review and meta-analysis evaluating the effectiveness of indocyanine green (ICG) fluorescence imaging in preventing postoperative bile leakage after hepatectomy.

Postoperative bile leakage is a clinically significant complication that can lead to substantial morbidity and even mortality. Therefore, this study addresses an important and clinically relevant question.

In addition, the protocol is developed in accordance with PRISMA-P guidelines, and the prospective publication of the protocol enhances transparency and methodological rigor. Overall, the manuscript is well structured and of clear clinical interest.

I have a few minor comments that may help further strengthen the clarity and robustness of the protocol:

1. Introduction (clinical impact of bile leakage)

The Introduction provides a general overview of postoperative bile leakage; however, the clinical impact could be described in greater detail.

It would strengthen the rationale if the authors could include more specific information, supported by references, regarding:

• the association between bile leakage and postoperative mortality, and

• its impact on prolonged hospitalization and healthcare burden.

Providing quantitative context would help emphasize the importance of this study.

2. Definition of ICG administration purpose (P7, L123–)

In the section describing the definitions of ICG administration routes, it may be important to further clarify the intended purpose of ICG use.

Specifically, studies in which ICG is administered:

• primarily for bile leak detection, versus

• for other purposes (e.g., tumor localization or negative staining for anatomical resection), with bile leak detection assessed as a secondary outcome

may differ substantially in how bile leakage is visualized.

For example, in cases involving negative staining during anatomical resection, bile leakage from the transection plane may be less detectable using ICG. This difference in intended use may systematically bias the sensitivity of bile leak detection and contribute to heterogeneity in the results.

Therefore, further clarification or stratification based on the primary intent of ICG use would strengthen the methodological consistency of the review.

3. Clarification of “hepatectomy-specific data” (P12, L94)

The term “hepatectomy-specific data” is somewhat unclear.

It would be helpful if the authors could explicitly define what is considered “hepatectomy-specific,” particularly in studies involving mixed surgical populations.

4. Surgical approach (anatomical vs non-anatomical resection) (P17, L289–)

The incidence and severity of bile leakage may differ between:

• anatomical hepatectomy (e.g., lobectomy or segmentectomy), and

• non-anatomical (parenchymal-sparing) resection.

This factor may influence the outcomes and should be considered, for example, in subgroup analyses or in the interpretation of findings.

5. Details of narrative synthesis

The manuscript is generally well described; however, given the anticipated heterogeneity, it is likely that some outcomes will require narrative synthesis rather than quantitative meta-analysis.

In this context, it would improve methodological transparency if the authors could provide more detailed information on:

• how studies will be grouped,

• how the direction and magnitude of effects will be summarized, and

• how conclusions (particularly for the primary outcome) will be derived in the absence of quantitative pooling.

In addition, the use of a structured approach to narrative synthesis (e.g., following SWiM guidance or using effect direction plots) may enhance reproducibility and reduce potential interpretive bias.

In summary, this is a well-designed and clinically meaningful protocol addressing an important topic. The methodology is generally sound, and the manuscript is clearly written.

Addressing the points above would further improve clarity and methodological transparency.

I therefore recommend minor revision.

Reviewer #7: I read with great interest the protocol for a systematic review by Hanaki et al. The protocol is described in detail and pertains to relevant topic of ICG guided detection of bile leaks during hepatectomy. Even though the manuscript is well written, I will be more interested in the results of this systematic review and meta-analysis. I would suggest authors to make the manuscript concise as there are lot of repetitions.

Reviewer #8: The overall protocol is well-designed, logically complete, and academically feasible, but there is still room for improvement. Specific comments are as follows:

1. Study Population and Exclusion Criteria

The inclusion/exclusion boundary for mixed hepatobiliary procedures and extrahepatic bile duct resection is ambiguous, which may lead to confusion in data extraction.

2. Definition of Intervention and Comparator

Standards for ICG dosage, concentration, and timing of administration are not unified. The methods of “conventional leak testing” in the comparator group vary widely, which may introduce heterogeneity.

Regarding the mapping rule for “clinically relevant bile leakage” (primary outcome)

The protocol stipulates that when ISGLS grading is not reported in the original studies, “requirement for clinical intervention (e.g., ERCP, drainage, reoperation)” should be used to map to Grade ≥B. This may lead to false-positive mapping. The core of ISGLS Grade B is “necessity of active therapeutic intervention”, whereas some Grade A bile leaks (only detected radiologically without changing the clinical pathway) may also receive passive prophylactic drainage. Directly equating to “drainage” may overestimate the incidence rate.

Revise the mapping rule to “explicit description of the need for/implementation of therapeutic intervention due to bile leakage (e.g., therapeutic ERCP, percutaneous drainage, reoperation) that goes beyond routine postoperative care or prophylactic drainage”. Meanwhile, add an item in the data extraction form: “whether a causal relationship between intervention and bile leakage is clearly stated”.

3. Outcome Measures and Conversion Rules

The equivalent conversion rule for missing ISGLS grading is excessively broad and may lead to misclassification of outcomes.

Regarding subgroup analysis

Using “minimally invasive surgery (MIS)” as a subgroup is appropriate. However, the value of ICG fluorescence imaging, surgical exposure, and imaging time after ICG administration may differ substantially between laparoscopic/robotic surgery and open surgery. The current protocol does not further distinguish “total robotic”, “laparoscopic”, and “robot‑assisted” procedures within the MIS group.

In subgroup analysis, if data permit, at least distinguish “pure laparoscopic/robotic” from “open” surgery. Acknowledge in the discussion that subgroup results may have interaction effects because fluorescence imaging may have higher sensitivity under MIS conditions (close lens, low-light environment).

4. Discussion Section

The clinical implications of positive/negative results and directions for future research are not pre-specified.

Add the following content: If the results are positive, routine intraoperative intrabiliary ICG administration for bile leak detection is recommended; if negative, current evidence is insufficient and large‑sample RCTs are needed. Clearly state the limitations: variations in administration routes, lack of dose–response relationship, and predominance of single‑center studies.

Reviewer #9: This study is a protocol for a systematic review and meta-analysis evaluating whether intraoperative indocyanine green (ICG) fluorescence imaging-guided bile leak detection, followed by immediate repair or reinforcement, reduces clinically relevant postoperative bile leakage after hepatectomy, namely ISGLS grade B/C bile leakage. The authors plan to include randomized controlled trials and comparative nonrandomized studies. The primary outcome is ISGLS grade B/C bile leakage, and secondary outcomes include any bile leakage, bile leak-related interventions, major postoperative complications, postoperative length of hospital stay, and mortality. The planned classification of ICG administration routes into intravascular, intrabiliary, and mixed administration, the separate synthesis of RCTs and NRSI, and the use of RoB 2, ROBINS-I, and GRADE are generally appropriate.

However, the current manuscript does not sufficiently explain its originality as a synthesis focused specifically on ICG and clinically relevant bile leakage. Meta-analyses of intraoperative bile leak tests in liver resection already exist. For example, the 2019 HPB review included eight studies and reported that intraoperative bile leak testing was associated with reductions in postoperative bile leakage, overall complications, reintervention, and length of hospital stay. In addition, several classical and directly relevant studies on ICG-based bile leak detection can be identified, including Sakaguchi et al. 2010, Kaibori et al. 2011, and Hanaki et al. 2022/2025. However, at least Sakaguchi 2010, Hanaki 2022 in Anticancer Research, and Vaska 2019 in HPB do not appear to be included in the manuscript’s reference list. The background section and the explanation of novelty therefore need to be reconstructed.

Major comments

1. The relationship with previous reviews of intraoperative bile leak tests in general is insufficiently explained. Although Wang 2013 is cited, the systematic review and meta-analysis by Vaska 2019 in HPB does not appear to be cited. Vaska 2019 addressed bile leak tests after liver resection in general and included ICG and the white test among its search terms. This review should be positioned as an important direct predecessor of the present manuscript. The authors should clearly differentiate the present review by stating that previous reviews assessed bile leak tests in general, whereas the present study focuses specifically on ICG fluorescence imaging, uses ISGLS grade B/C bile leakage as the primary outcome, and evaluates effects according to the route of ICG administration.

2. Several directly relevant studies on ICG-based bile leak detection should be addressed. Sakaguchi et al. 2010, “Bile leak test by indocyanine green fluorescence images after hepatectomy,” is a foundational paper on ICG-based bile leak testing after hepatectomy and should be mentioned in the Introduction. In addition, Hanaki et al. 2022 in Anticancer Research, “Surgical Administration of Indocyanine Green in Hepatectomy for Improved Bile Leakage Detection,” is likely to include comparative data from the authors’ own group and is important both for the background of this review and for managing potential overlapping cohorts. Although Kaibori 2011 and Hanaki 2025 are cited, the protocol should explicitly state how the authors will handle possible patient or institutional overlap among these studies, Hanaki 2022, the Hanaki 2023 protocol, and related case reports.

3. Confounding in nonrandomized studies requires more detailed prespecification. In nonrandomized studies, especially those using historical controls, confounding is likely to be substantial. Potential confounders include surgical period, surgeon experience, institutional practice, drain placement policy, implementation of ERAS pathways, major versus minor hepatectomy, cirrhosis, cholestasis, biliary reconstruction, surgical procedure, tumor type, and history of chemotherapy. ROBINS-I is a tool for assessing bias in comparative effectiveness estimates from nonrandomized intervention studies, and its appropriate use requires prior specification of the target trial, confounding domains, co-interventions, and the approach to selection bias assessment. The authors should not merely state that adjusted estimates will be preferred. They should specify which confounders will be considered minimally necessary, how adjusted ORs, RRs, and HRs will be handled on a common effect scale, and how they will avoid mixing adjusted and unadjusted estimates in the same synthesis.

4. The eligibility criteria for biliary reconstruction and mixed hepatobiliary or pancreatic procedures should be clarified. The manuscript focuses on bile leakage from the liver transection plane or biliary stump after hepatectomy. However, although biliary reconstruction is listed as an exclusion criterion, the protocol also states that mixed cohorts may be included in sensitivity analyses or narrative synthesis when separate data are unavailable. This compromise is understandable, but bile leakage arising from biliary reconstruction or pancreatic surgery differs in mechanism, risk profile, and diagnosis from leakage from the hepatic transection plane. Such studies should not be pooled with studies of bile leakage from the liver transection surface. One of the cited references concerns ICG use for hepaticojejunostomy in robot-assisted pancreatic surgery. This may be acceptable as background literature, but the authors should clearly state that it is not direct evidence for the target condition of this review.

Minor comments

1. If major hepatectomy is used as a subgroup variable, the authors should prespecify its definition, for example, resection of three or more sections or three or more Couinaud segments.

2. The authors should clarify whether laparoscopic and robotic surgery will be grouped together as minimally invasive surgery or, if feasible, analyzed separately. Camera performance, field of view, and overlay functions for ICG imaging may differ across surgical platforms.

Reviewer #10: Bile leakage is one of the unpleasant postoperative complications that would be avoided in liver surgery. Once bile leakage occurs after liver surgery, a great deal of mental and physical stresses would be required for both the patients and surgeons. Liver surgeons have therefore made efforts to reduce the occurrence of bile leakage, it is however difficult to make zero its morbidity. At present, the intraoperative detection of bile leakage using indocyanine green (ICG) might be innovative and the authors have continued to make improvement and reports for this technique. In this study, the authors made a plan for the systematic review and meta-analysis for the detection of bile leakage using ICG. The reviewer has an interest for this report, there are however some confirmations and questions to clear up more as below:

#1: It is certainly that there were few reports of systematic review and meta-analysis for the intraoperative detection of bile leakage using ICG. On the other hand, the number of papers on this topic itself is however not particularly large. In this restrictive topic, the reviewer doubts the necessity and meaning for systematic review and meta-analysis.

Reviewer #11: This study is actually a protocol to perform a study and depicts the authors' submission for publication of their desired PRISMA platform to investigate the benefits and best practices of utilizing ICG for detecting biliary leaks for hepatic resections. The concepts are all very good however it is unclear to the reader initially that there would be no reporting of outcomes or results of their analyses. I think if the authors would clarify to make that clear they are only intending to publish their methods for this analysis, it would provide more clarity to the reader

**********

7. PLOS authors have the option to publish the peer review history of their article (what does this mean?). If published, this will include your full peer review and any attached files.

If you choose “no”, your identity will remain anonymous but your review may still be made public.

**Do you want your identity to be public for this peer review?**  For information about this choice, including consent withdrawal, please see our Privacy Policy.

Reviewer #1: No

Reviewer #2: **Yes:** Rezende, Lucas

Reviewer #3: No

Reviewer #4: **Yes:** Muhammad N. Khan

Reviewer #5: No

Reviewer #6: **Yes:** Hideyuki Yoshitomi

Reviewer #7: No

Reviewer #8: No

Reviewer #9: No

Reviewer #10: No

Reviewer #11: **Yes:** David Mulligan, MD

**********

[NOTE: If reviewer comments were submitted as an attachment file, they will be attached to this email and accessible via the submission site. Please log into your account, locate the manuscript record, and check for the action link "View Attachments". If this link does not appear, there are no attachment files.]

To ensure your figures meet our technical requirements, please review our figure guidelines: https://journals.plos.org/plosone/s/figures

You may also use PLOS’s free figure tool, NAAS, to help you prepare publication quality figures: https://journals.plos.org/plosone/s/figures#loc-tools-for-figure-preparation.

NAAS will assess whether your figures meet our technical requirements by comparing each figure against our figure specifications.

# Author comment: Author response to Decision Letter 1

**DOI:** 10.1371/journal.pone.0346089.r002

11 Jun 2026

Thank you for considering our manuscript for publication in PLOS ONE. We are grateful for the time and effort the editors and reviewers have devoted to evaluating our work. In response to their valuable feedback, we have revised the manuscript and believe it has been substantially improved. Our point-by-point responses to all comments are provided below. (All responses in this comments sheet are shown in GREEN text.)

-----------

This decision reflects the overall merit of the work. Several reviewers accepted the manuscript without reservation, commending its PRISMA-P compliance, pre-registration, and clinical relevance. But a number of substantive methodological concerns must be addressed before the manuscript can be accepted.

The required revisions are as follows:

1. ROBINS-I should be replaced with ROBINS-E, which is the current methodological standard for this type of bias assessment.

We thank the Academic Editor for this important methodological comment. We agree that the risk-of-bias tool should be current and appropriate for the type of evidence being assessed.

Before finalizing the revision, we sought clarification from the PLOS ONE Editorial Office regarding whether ROBINS-E or an intervention-focused tool would be more appropriate for this review. We explained that the review question concerns the comparative effect of an intraoperative intervention, namely ICG fluorescence imaging-guided bile leak detection, rather than the effect of an exposure. The Associate Editor agreed that a tool designed to assess interventional studies is most appropriate and recommended specifying ROBINS-I V2 in the assessment plan.

Accordingly, we have revised the “Risk of bias assessment” section to specify that comparative nonrandomized studies will be assessed using ROBINS-I V2. We also added a brief justification in the protocol, stating that ROBINS-I V2 was selected because this review evaluates the comparative effect of an intraoperative intervention rather than an exposure. In addition, we strengthened the prespecified target trial framework, minimally required confounding domains, relevant co-interventions, and the handling of adjusted and unadjusted estimates.

2. The ISGLS mapping rule for studies not reporting standardized grading requires tightening. The current rule risks overestimating Grade B leakage by equating any drainage with therapeutic intervention. Please revise to require explicit evidence of therapeutic intent beyond routine or prophylactic drainage, and add a corresponding item to the data extraction form.

We thank the Academic Editor for this important comment. We agree that the previous mapping rule could have been interpreted too broadly, particularly if drainage was considered equivalent to ISGLS grade B leakage without clear evidence of therapeutic intent.

We have revised the “Primary outcome” section to tighten the mapping rule for studies that do not report ISGLS grading. In the revised protocol, non-ISGLS outcome definitions will be mapped to Grade ≥B-equivalent only when the report explicitly states that therapeutic intervention or treatment escalation was required or performed because of postoperative bile leakage and that the intervention went beyond routine postoperative care or prophylactic drainage. We also clarified that routine drain placement, prophylactic drainage, radiological detection without a change in management, or persistent drainage without explicit therapeutic intent will not be mapped as Grade ≥B-equivalent.

In addition, we revised the “Selection process and data extraction” section to add corresponding extraction items. We will now extract whether therapeutic intervention or treatment escalation was explicitly attributed to bile leakage and whether the intervention went beyond routine postoperative care or prophylactic drainage. If therapeutic intent or attribution to bile leakage cannot be determined because of insufficient reporting, the study will be excluded from the meta-analysis of the primary outcome but may still contribute to secondary outcome analyses or narrative synthesis where applicable.

3. The background section should be substantially revised to position the present review relative to Vaska et al. (2019, HPB), which assessed intraoperative bile leak tests after liver resection in general and represents the most directly relevant predecessor. The novelty of the present review, i.e., its exclusive focus on ICG fluorescence imaging, use of ISGLS Grade B/C as the primary outcome, and stratification by administration route, should be clearly articulated.

We thank the Academic Editor for this important comment. We revised the Introduction to position the systematic review and meta-analysis by Vaska et al. as the most directly relevant predecessor on intraoperative bile leak testing after liver resection in general. We also clarified that the present review differs from previous reviews by focusing specifically on ICG fluorescence imaging-guided bile leak detection, using clinically relevant ISGLS grade B/C bile leakage as the primary outcome, and exploring heterogeneity according to the route of ICG administration. Vaska et al. has now been added as reference [9].

4. Sakaguchi et al. (2010) and Hanaki et al. (2022, Anticancer Research) should be cited and discussed. The protocol must explicitly prespecify how potential patient or institutional cohort overlap across these studies, the Hanaki 2023 protocol, and related case reports will be identified and handled.

We thank the Academic Editor for this important comment. We revised the Introduction to cite and discuss Sakaguchi et al. as an early clinical study of ICG fluorescence-based bile leak testing after hepatectomy and Hanaki et al. 2022 in Anticancer Research as a directly relevant study evaluating ICG fluorescence-guided bile leak detection. These studies are now cited in the Introduction as references [14] and [17], respectively; the relevant sentence now cites subsequent studies of ICG fluorescence-guided bile leak detection using intrabiliary or systemic administration [13, 15-17].

We agree with the Academic Editor that potential patient or institutional cohort overlap should be prespecified. We have added a new description to the “Selection process and data extraction” section. In the revised protocol, we state that possible overlap among multiple reports from the same or potentially overlapping cohort will be assessed by comparing the institution, author group, enrolment period, study design, intervention protocol, sample size, ethics approval or trial registration information, and patient characteristics. We also state that the possibility of overlap and the rationale for selecting one report over another will be recorded in the data extraction form. When overlap is suspected, the most complete comparative dataset that is most appropriate for the outcome of interest will be prioritized for quantitative synthesis, and protocols, case reports, video reports, and overlapping descriptive reports will not be double-counted in any meta-analysis.

5. Confounding variables for nonrandomized studies must be prespecified in greater detail. The authors should identify the minimally required confounders for ROBINS-E assessment and specify how adjusted and unadjusted estimates will be handled to avoid inappropriate mixing in the same synthesis.

We thank the Academic Editor for this important comment. We have revised the Methods to prespecify the handling of confounding in comparative nonrandomized studies in greater detail.

In the “Selection process and data extraction” section, we added that adjusted effect estimates, the effect measure used, and the covariates included in the adjustment model will be extracted when available. We also added that information on prespecified confounding domains will be extracted, including surgical period, surgeon or institutional experience, institutional perioperative practice including enhanced recovery pathways and drain management policy, extent of hepatectomy, surgical approach, background liver disease or cirrhosis, cholestasis or biliary obstruction, biliary reconstruction or extrahepatic bile duct resection, surgical indication or tumor type, and history of preoperative chemotherapy.

In the “Risk of bias assessment” section, we added that the assessment of comparative nonrandomized studies will follow a prespecified target trial framework. The target trial is conceptualized as a comparison between intraoperative ICG fluorescence imaging-guided bile leak detection and conventional intraoperative assessment without ICG fluorescence imaging among patients undergoing hepatectomy. The minimally required confounding domains and relevant co-interventions are now explicitly described.

In the “Data synthesis” section, we clarified that adjusted effect estimates will be prioritized when available and synthesized using the generic inverse variance approach. We also clarified that adjusted odds ratios, risk ratios, and hazard ratios will be extracted on the logarithmic scale with their standard errors when reported or calculable. Because these effect measures are not directly interchangeable, they will not be combined in the same pooled analysis unless a clinically and statistically justified conversion is possible. We further clarified that adjusted and unadjusted estimates will not be pooled together in the same primary synthesis. When adjusted estimates are unavailable, unadjusted event data will be analyzed separately as an exploratory or sensitivity analysis, and the limitations of residual confounding will be explicitly considered.

6. The inclusion of mixed hepatobiliary and biliary reconstruction procedures requires clearer boundaries. Studies involving bile leakage from biliary anastomoses or pancreatic surgery should not be pooled with hepatic transection plane leakage studies. A concrete, quantitative decision rule for inclusion of mixed-procedure cohorts should be provided.

We thank the Academic Editor for this important comment. We agree that bile leakage from biliary-enteric anastomoses or pancreatic surgery differs mechanistically from bile leakage arising from the hepatic transection plane or biliary stump and should not be pooled with the target condition of this review.

We have revised the “Population” and “Exclusion criteria” sections to clarify the boundaries for mixed hepatobiliary and mixed surgical cohorts. In the revised protocol, hepatectomy-specific data are defined as outcome data reported separately for patients undergoing liver resection in whom postoperative bile leakage is attributable to the hepatic transection plane and/or biliary stump, rather than to biliary-enteric anastomosis, pancreatic surgery, or other non-hepatectomy procedures.

We also clarified that mixed hepatobiliary or mixed surgical cohorts will be eligible for primary quantitative synthesis only when outcome data for eligible hepatectomy patients without biliary reconstruction are separately reported or can be derived from the publication. If hepatectomy-specific outcome data cannot be separated, the study will not be included in the primary quantitative synthesis. We decided not to apply a proportion-based threshold for nonseparable mixed cohorts, because doing so could still combine mechanistically different types of bile leakage. Instead, we prespecified a stricter rule requiring separability of hepatectomy-specific outcomes for inclusion in quantitative synthesis. Studies focused on pancreatic surgery or hepaticojejunostomy-related leakage will be used only as background literature or narrative context, where relevant, and will not be eligible for quantitative synthesis of bile leakage from the hepatic transection plane or biliary stump after hepatectomy.

7. The primary intent of ICG administration (bile leak detection vs. tumor localization or anatomical resection) should be addressed as a potential source of systematic heterogeneity, with clarification or stratification proposed.

We thank the Academic Editor for this important comment. We revised the “Intervention” section to clarify how the primary intent of ICG administration will be handled. In the revised protocol, included studies will be classified as primary bile leak detection, multipurpose or other primary fluorescence-guided intent with explicit bile leak assessment, or unclear intent. Studies without any explicit intent to detect bile leakage or guide repair will be excluded.

We also revised the “Subgroup analyses” section to include primary intent of ICG administration as a prespecified source of heterogeneity. When data permit, studies will be compared according to primary bile leak detection versus multipurpose or other primary fluorescence-guided intent with explicit bile leak assessment; studies with unclear intent will be excluded from this subgroup analysis and summarized descriptively.

8. An operational definition of "hepatectomy" should be provided in the eligibility criteria, and the definition of major hepatectomy used in subgroup analyses should be prespecified.

We thank the Academic Editor for this important comment. We have revised the “Population” section of the eligibility criteria to provide an operational definition of hepatectomy. In the revised protocol, hepatectomy is defined as surgical resection of liver parenchyma involving a transection plane, including wedge resection, partial hepatectomy, segmentectomy, sectionectomy, hemihepatectomy, and extended hepatectomy. We also clarified that procedures without liver parenchymal resection, such as ablation alone or nonresectional biliary procedures, will not be considered hepatectomy.

We also revised the “Subgroup analyses” section to prespecify the definition of major hepatectomy. For the purpose of subgroup analysis in this review, major hepatectomy will be defined as resection of three or more Couinaud segments, and minor hepatectomy will be defined as resection of fewer than three Couinaud segments.

9. The manuscript should be revised for conciseness, removing repetitive passages.

We thank the Academic Editor for this helpful comment. We revised the manuscript to reduce repetitive descriptions and improve readability while retaining the methodological details required for a protocol manuscript. In particular, we shortened the Discussion by removing repeated descriptions of the primary outcome, ISGLS mapping, subgroup analyses, and separate synthesis of randomized and nonrandomized studies, which are already described in detail in the Methods. The revised Discussion now focuses more concisely on the rationale, anticipated heterogeneity, limitations, and implications of the planned review.

The following points require clarification but are of lesser priority:

• Whether laparoscopic and robotic approaches will be analyzed separately within the minimally invasive surgery subgroup.

We thank the Academic Editor for this helpful clarification. We revised the “Subgroup analyses” section to specify how minimally invasive approaches will be handled. In the revised protocol, surgical approach will first be categorized as open versus minimally invasive surgery. When data permit, minimally invasive surgery will be further categorized as pure laparoscopic, robotic, or robot-assisted/hybrid approaches. These categories will be examined descriptively and, if sufficient comparative data are available, in exploratory subgroup analyses.

• How studies with shorter or variable follow-up will be handled relative to the 90-day primary outcome window.

We thank the Academic Editor for this helpful clarification. We revised the “Primary outcome” section to clarify the handling of shorter or variable follow-up windows. When multiple follow-up time points are reported, we will use the longest follow-up within 90 days postoperatively; if no time point falls within 90 days, we will use the time point closest to 90 days. For studies reporting in-hospital, 30-day, 90-day, or otherwise variable follow-up windows, the exact follow-up window will be extracted and considered when assessing heterogeneity. Studies with unclear or insufficiently reported follow-up windows will be included in the primary outcome meta-analysis only when outcom

Attachment Submitted filename: 260612 Response to reviwers and editors.docx

# Peer review: Decision Letter 1

**DOI:** 10.1371/journal.pone.0346089.r003

**Authors:** Athanasios Pantelis, Athanasios Pantelis

22 Jun 2026

ICG fluorescence imaging-guided bile leak detection to reduce clinically relevant bile leakage after hepatectomy: a protocol for a systematic review and meta-analysis

PONE-D-26-12531R1

Dear Dr. Hanaki,

We’re pleased to inform you that your manuscript has been judged scientifically suitable for publication and will be formally accepted for publication once it meets all outstanding technical requirements.

Within one week, you’ll receive an e-mail detailing the required amendments. When these have been addressed, you’ll receive a formal acceptance letter and your manuscript will be scheduled for publication.

An invoice will be generated when your article is formally accepted. Please note, if your institution has a publishing partnership with PLOS and your article meets the relevant criteria, all or part of your publication costs will be covered. Please make sure your user information is up-to-date by logging into Editorial Manager at Editorial Manager® and clicking the ‘Update My Information' link at the top of the page. For questions related to billing, please contact billing support.

If your institution or institutions have a press office, please notify them about your upcoming paper to help maximize its impact. If they’ll be preparing press materials, please inform our press team as soon as possible -- no later than 48 hours after receiving the formal acceptance. Your manuscript will remain under strict press embargo until 2 pm Eastern Time on the date of publication. For more information, please contact onepress@plos.org.

Kind regards,

Athanasios G. Pantelis

Academic Editor

PLOS One

Additional Editor Comments (optional):

Reviewers' comments:

Reviewer's Responses to Questions

**Comments to the Author**

1. Does the manuscript provide a valid rationale for the proposed study, with clearly identified and justified research questions?

The research question outlined is expected to address a valid academic problem or topic and contribute to the base of knowledge in the field.

Reviewer #1: Yes

Reviewer #3: Yes

Reviewer #5: Yes

Reviewer #8: Yes

**********

2. Is the protocol technically sound and planned in a manner that will lead to a meaningful outcome and allow testing the stated hypotheses?

The manuscript should describe the methods in sufficient detail to prevent undisclosed flexibility in the experimental procedure or analysis pipeline, including sufficient outcome-neutral conditions (e.g. necessary controls, absence of floor or ceiling effects) to test the proposed hypotheses and a statistical power analysis where applicable. As there may be aspects of the methodology and analysis which can only be refined once the work is undertaken, authors should outline potential assumptions and explicitly describe what aspects of the proposed analyses, if any, are exploratory.

Reviewer #1: Yes

Reviewer #3: Yes

Reviewer #5: Yes

Reviewer #8: Yes

**********

3. Is the methodology feasible and described in sufficient detail to allow the work to be replicable?

Descriptions of methods and materials in the protocol should be reported in sufficient detail for another researcher to reproduce all experiments and analyses. The protocol should describe the appropriate controls, sample size calculations, and replication needed to ensure that the data are robust and reproducible.

Reviewer #1: Yes

Reviewer #3: Yes

Reviewer #5: Yes

Reviewer #8: Yes

**********

4. Have the authors described where all data underlying the findings will be made available when the study is complete?

The PLOS Data policy requires authors to make all data underlying the findings described in their manuscript fully available without restriction, with rare exception, at the time of publication. The data should be provided as part of the manuscript or its supporting information, or deposited to a public repository. For example, in addition to summary statistics, the data points behind means, medians and variance measures should be available. If there are restrictions on publicly sharing data—e.g. participant privacy or use of data from a third party—those must be specified.

Reviewer #1: Yes

Reviewer #3: Yes

Reviewer #5: Yes

Reviewer #8: Yes

**********

5. Is the manuscript presented in an intelligible fashion and written in standard English?

PLOS ONE does not copyedit accepted manuscripts, so the language in submitted articles must be clear, correct, and unambiguous. Any typographical or grammatical errors should be corrected at revision, so please note any specific errors here.

Reviewer #1: Yes

Reviewer #3: Yes

Reviewer #5: Yes

Reviewer #8: Yes

**********

6. Review Comments to the Author

Please use the space provided to explain your answers to the questions above and, if applicable, provide comments about issues authors must address before this protocol can be accepted for publication. You may also include additional comments for the author, including concerns about research or publication ethics.

You may also provide optional suggestions and comments to authors that they might find helpful in planning their study.

(Please upload your review as an attachment if it exceeds 20,000 characters)

Reviewer #1: The revised manuscript remains of high quality, and the changes made in response to the other reviewers have further strengthened the paper. I have no further comments.

Reviewer #3: I appreciate the authors’ careful responses to the reviewers’ comments.

All of my concerns have been adequately addressed, and I have no further comments.

Reviewer #5: The protocol has been strengthened by the authors addressing the Reviewers' critiques. I have no further comments

Reviewer #8: The article has been revised very well. I really appreciate the efforts made by the authors for this. I suggest it be accepted for publication.

**********

7. PLOS authors have the option to publish the peer review history of their article (what does this mean?). If published, this will include your full peer review and any attached files.

If you choose “no”, your identity will remain anonymous but your review may still be made public.

**Do you want your identity to be public for this peer review?**  For information about this choice, including consent withdrawal, please see our Privacy Policy.

Reviewer #1: No

Reviewer #3: No

Reviewer #5: No

Reviewer #8: No

**********

# Editor report: Acceptance letter

**DOI:** 10.1371/journal.pone.0346089.r004

**Authors:** Athanasios Pantelis, Athanasios Pantelis

PONE-D-26-12531R1

PLOS One

Dear Dr. Hanaki,

I'm pleased to inform you that your manuscript has been deemed suitable for publication in PLOS One. Congratulations! Your manuscript is now being handed over to our production team.

At this stage, our production department will prepare your paper for publication. This includes ensuring the following:

* All references, tables, and figures are properly cited

* All relevant supporting information is included in the manuscript submission,

* There are no issues that prevent the paper from being properly typeset

You will receive further instructions from the production team, including instructions on how to review your proof when it is ready. Please keep in mind that we are working through a large volume of accepted articles, so please give us a few days to review your paper and let you know the next and final steps.

Lastly, if your institution or institutions have a press office, please let them know about your upcoming paper now to help maximize its impact. If they'll be preparing press materials, please inform our press team within the next 48 hours. Your manuscript will remain under strict press embargo until 2 pm Eastern Time on the date of publication. For more information, please contact onepress@plos.org.

You will receive an invoice from PLOS for your publication fee after your manuscript has reached the completed accept phase. If you receive an email requesting payment before acceptance or for any other service, this may be a phishing scheme. Learn how to identify phishing emails and protect your accounts at https://explore.plos.org/phishing.

If we can help with anything else, please email us at customercare@plos.org.

Thank you for submitting your work to PLOS ONE and supporting open access.

Kind regards,

PLOS ONE Editorial Office Staff

on behalf of

Dr. Athanasios G. Pantelis

Academic Editor

PLOS One
